# THADA Regulates the Organismal Balance between Energy Storage and Heat Production

**DOI:** 10.1016/j.devcel.2017.05.001

**Published:** 2017-05-22

**Authors:** Alexandra Moraru, Gulcin Cakan-Akdogan, Katrin Strassburger, Matilda Males, Sandra Mueller, Markus Jabs, Michael Muelleder, Martin Frejno, Bart P. Braeckman, Markus Ralser, Aurelio A. Teleman

## Main Text

(Developmental Cell *41*, 72–81; April 10, 2017)

The authors were kindly made aware by a reader that in Figure S3B, which shows expression of adh-GAL4, UAS-GFP in various tissues, an image showing expression in the gut was duplicated in the panel that should have shown the ovary. This mistaken duplication of the gut image has been replaced here and in the Supplemental Information PDF online with the correct image of the ovary from the same dataset. This correction does not change the conclusions of the paper, as both the gut and the ovary are GFP negative. The authors apologize for the error and any confusion it may have caused.

**Figure S3B:**
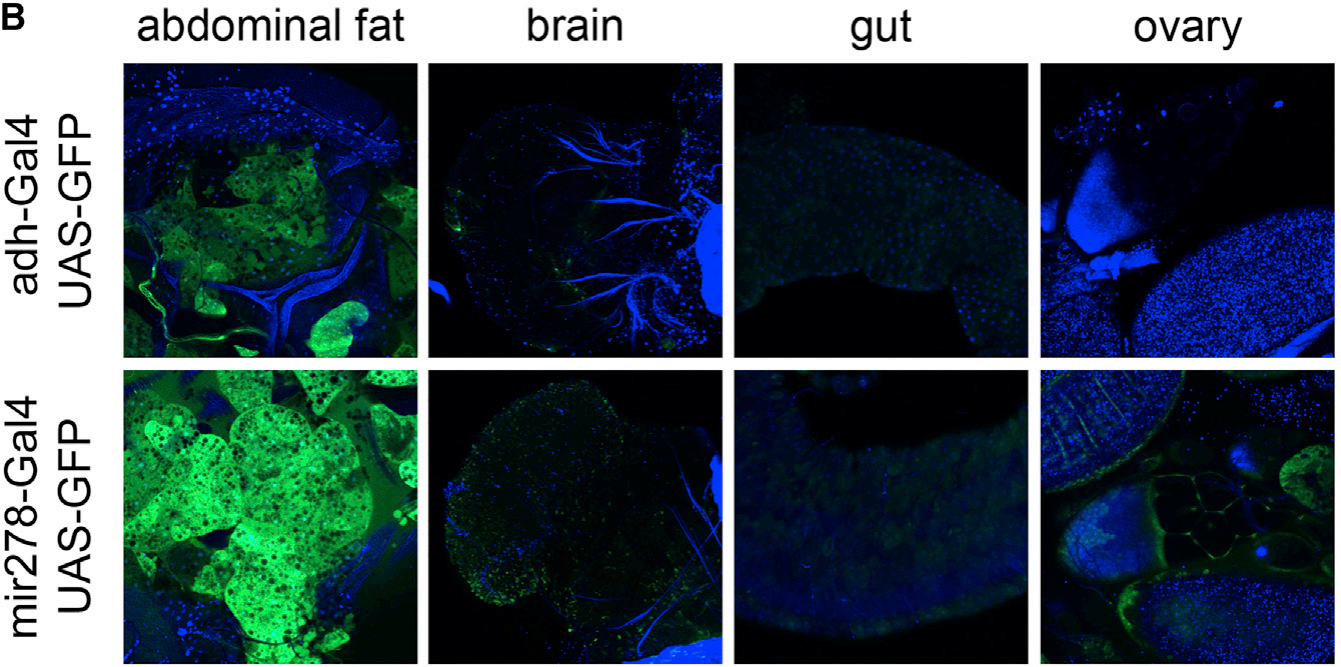
THADA Knockout Flies Do Not Have Ectopic Lipid Deposition (corrected)

**Figure S3B:**
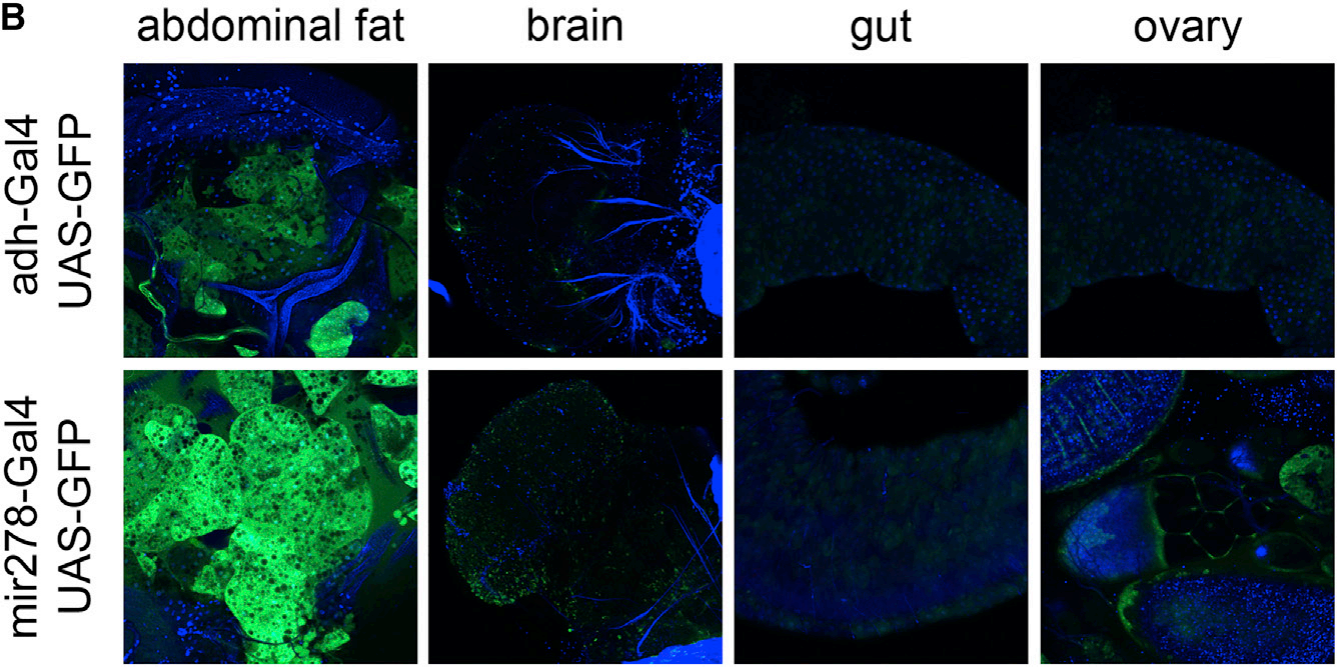
THADA Knockout Flies Do Not Have Ectopic Lipid Deposition (original)

