# Supplementary material for: THADA Regulates the Organismal Balance between Energy Storage and Heat Production
Source: Dev Cell. 2017 Apr 10;41(1):72–81.e6. doi: 10.1016/j.devcel.2017.03.016 (PMC5392496; doi:10.1016/j.devcel.2017.03.016)
Supplement: Document S1. Figures S1–S5 and Table S2 [file mmc1.pdf]

Developmental Cell, Volume *41*

Supplemental Information

THADA Regulates the Organismal Balance between Energy Storage and Heat Production

Alexandra Moraru, Gulcin Cakan-Akdogan, Katrin Strassburger, Matilda Males, Sandra Mueller, Markus Jabs, Michael Muelleder, Martin Frejno, Bart P. Braeckman, Markus Ralser, and Aurelio A. Teleman

Supplemental Information

Inventory

1. Supplemental Figures

   - Figure S1. (Related to main Figure 1).
   - Figure S2. (Related to main Figure 1).
   - Figure S3. (Related to main Figure 1).
   - Figure S4. (Related to main Figure 2).
   - Figure S5. (Related to main Figure 4).

2. Legends to Supplemental Figures

3. Supplemental Tables

   - Table S1. (Related to main Figure 2).
   - Table S2. (Related to STAR Methods).

Moraru et al. - Supplemental Figure 1

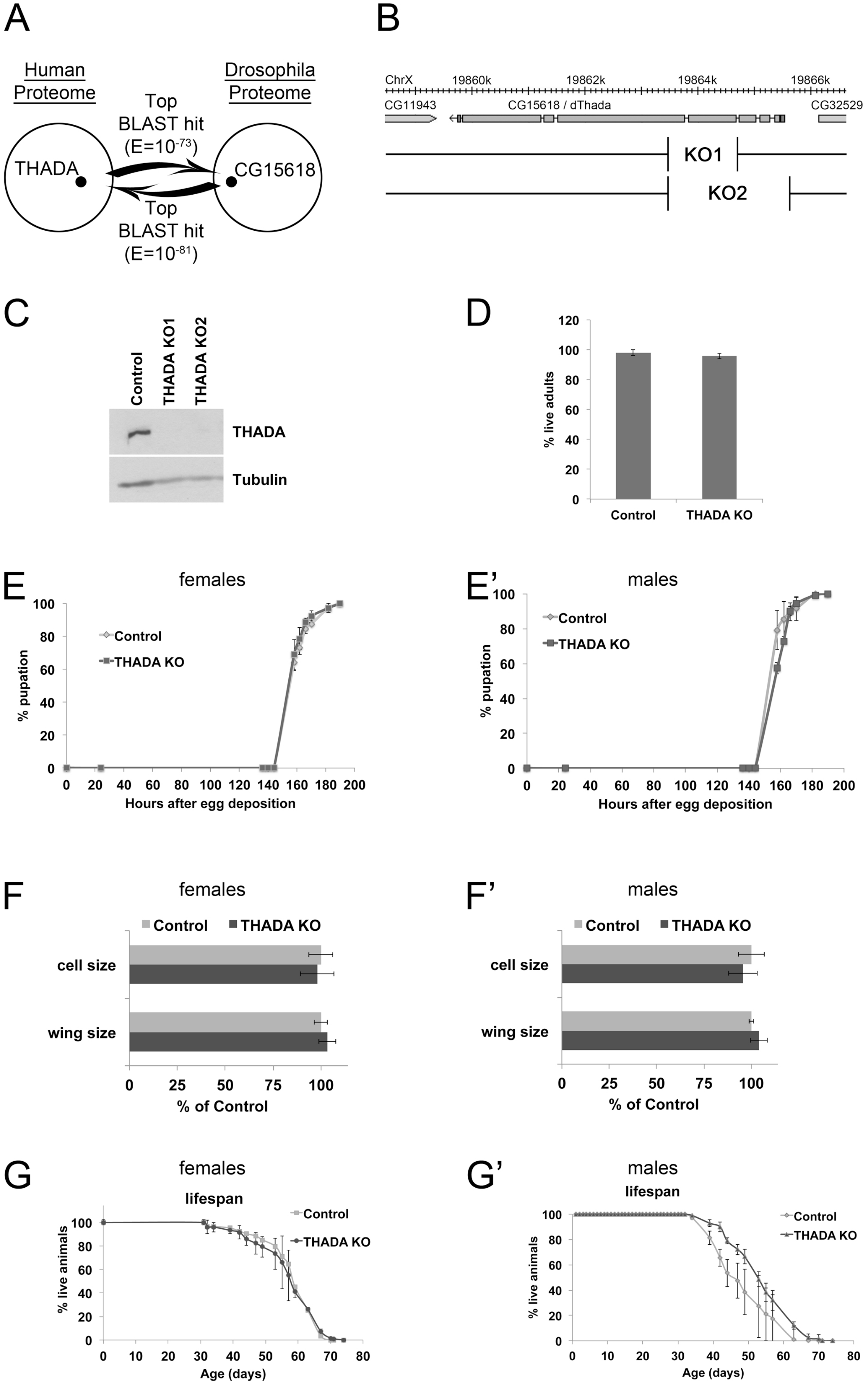

Moraru et al. - Supplemental Figure 2

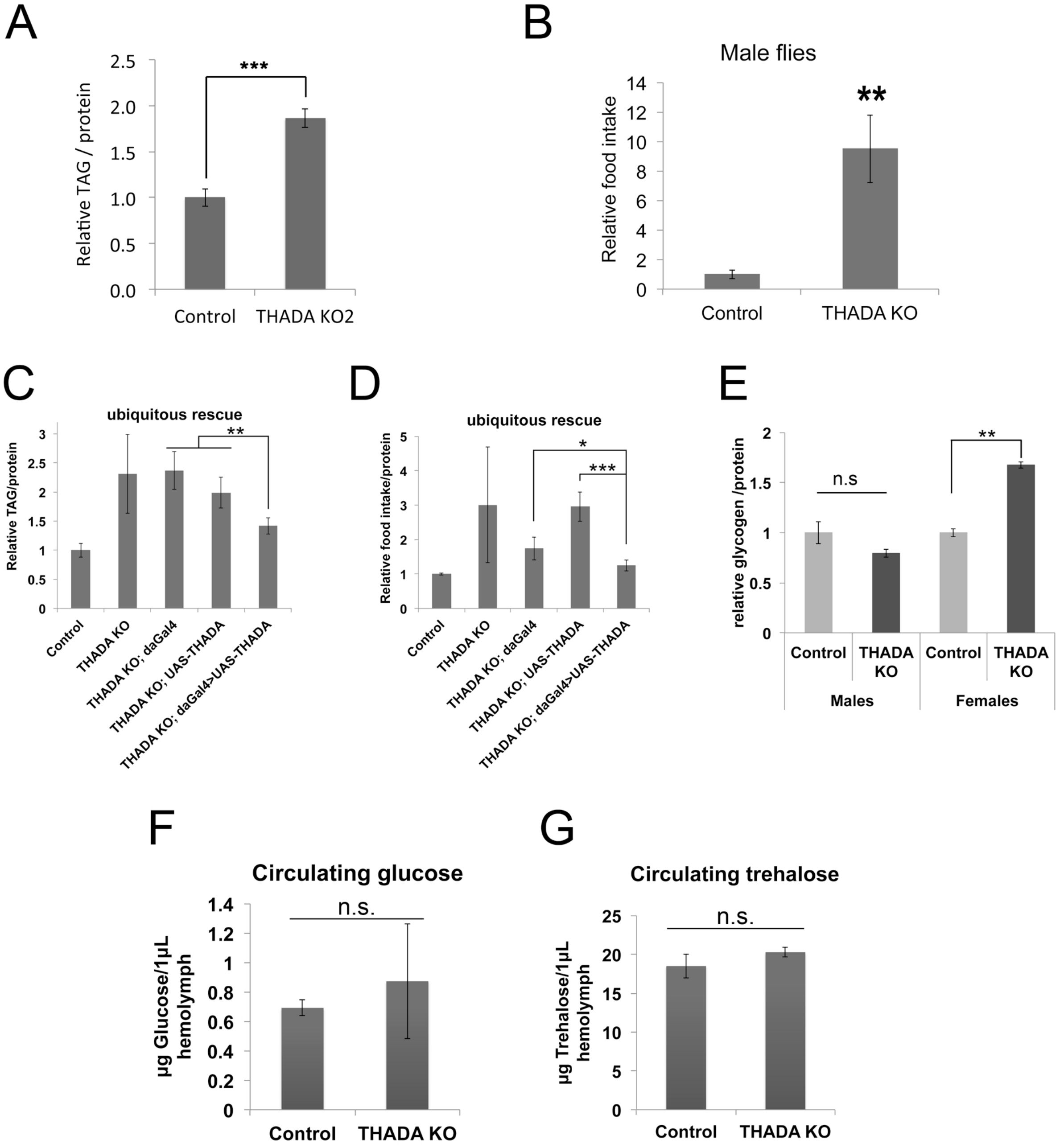

Moraru et al. - Supplemental Figure 3

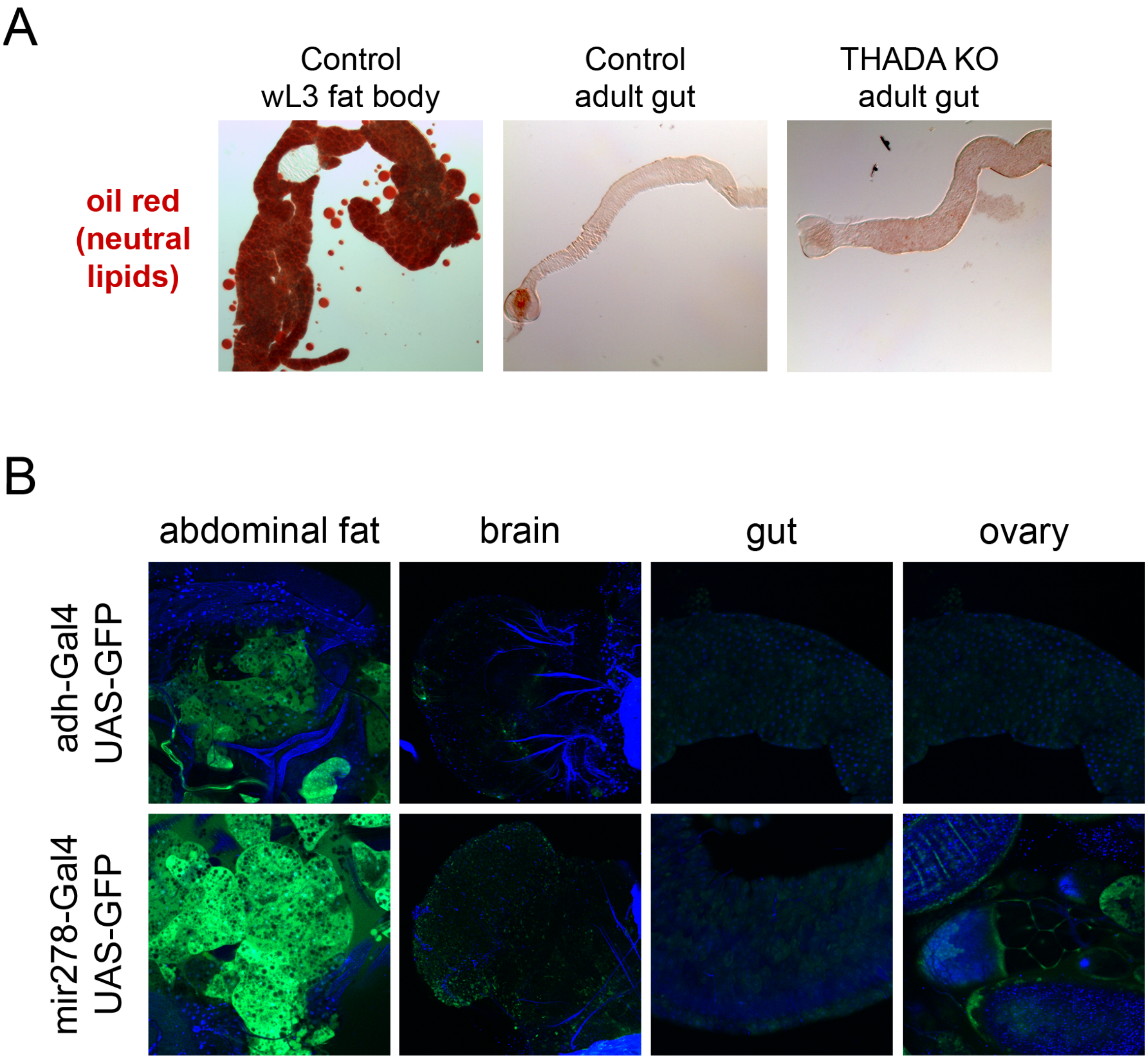

Moraru et al. - Supplemental Figure 4

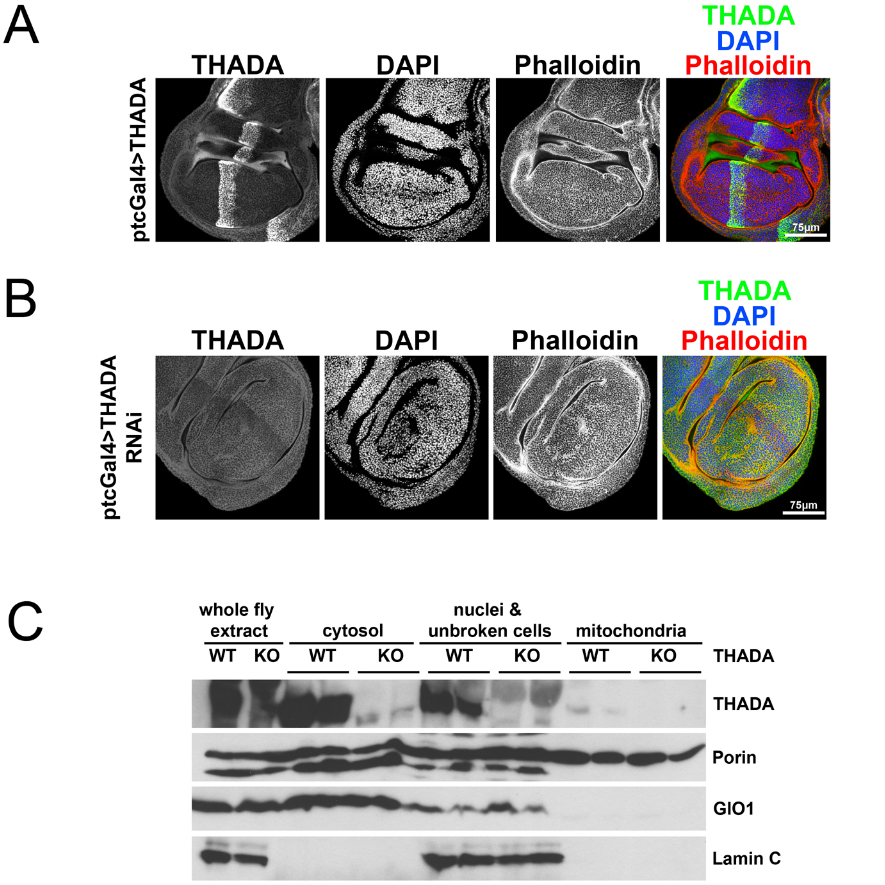

Moraru et al. - Supplemental Figure 5

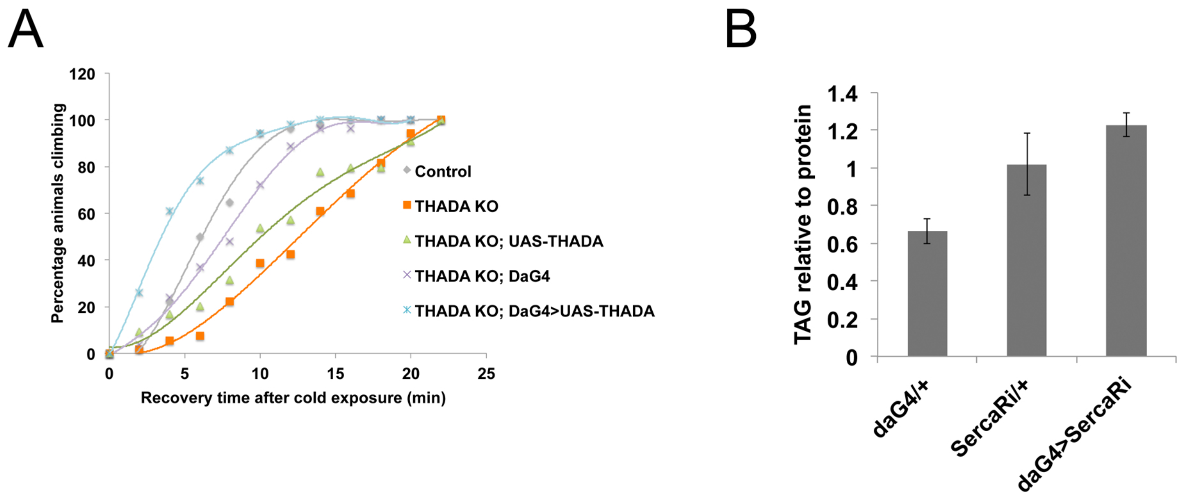

Legends to Supplemental Figures

Figure S1: *Drosophila THADA* knockout flies are viable. Related to Figure 1.

(A) CG15618 is the Drosophila ortholog of THADA. BLAST search (using the Flybase BLAST server) of the Drosophila proteome using the protein sequence of human THADA (NCBI Reference Sequence NP_071348.3) identifies CG15618 as the top hit, with an E value of 10^{-73}. Conversely, a BLAST search of the human proteome using the protein sequence of Drosophila CG15618 identifies THADA as the top hit with an E value of 10^{-81}, establishing an orthologous relationship between human THADA and Drosophila CG15618, which we henceforth refer to as Drosophila THADA.

(B) Schematic diagram of the *THADA/CG15618* genomic locus, indicating the knock-out regions.

(C) Anti-dTHADA antibody detects a band at the expected 220kDa size in control flies, but not in $THADA^{KO1}$ or $THADA^{KO2}$ flies.

(D-F') *THADA* knockout flies are viable (D), and have normal developmental timing (E-E') and size (F-F'). Sixty first-instar larvae were seeded per vial, and grown under controlled conditions. (D) Total number of eclosing adults was counted, and normalized to number of seeded larvae (n=3x60 animals). (E-E') Number of pupated females (E) or males (E') was quantified over time, represented as a percentage of total pupated animals (n=3x40 animals). (F-F') Total wing area and wing cell size (area per number of wing hairs) were measured and normalized to control animal values (n=8, representative of 3 experiments) for females (F) and males (F').

(G-G') *THADA*^{KO} flies have normal longevity. Lifespan curves of *THADA*^{KO} females (G) and males (G') are similar to those of controls. (n=4 x 35 to 55 adult females)

Figure S2: *Drosophila THADA* knockout flies have metabolic defects. Related to Figure 1.

(A) *THADA*^{KO2} animals, like *THADA*^{KO1} animals, have strongly elevated total body triglyceride levels, normalized to total body protein content. (n=3x8 3-day old adult females).

(B) *THADA*^{KO} males (like females, shown in Main Figure 1) are hyperphagic. Relative food intake in *THADA*^{KO} adult male flies, quantified by transferring flies from normal food to food containing Acid Blue 9 for one hour, followed by homogenization and measurement of OD625. (n=3x9)

(C-D) Ubiquitous expression of *THADA* from a transgene rescues the elevated triglycerides (C) and the hyperphagia (D) of *THADA*^{KO} flies. (C) Total body triglycerides normalize to total body protein. (D) Food intake quantified as in Main Figure 1B. Expression of *THADA* was achieved using the GAL4/UAS bipartite system whereby the GAL4 transcription factor activates expression downstream of a *UAS* enhancer element. Parental genotypes containing the *THADA* mutation and either the *GAL4* or *UAS-THADA* do not express *THADA*, whereas the combination (*THADA*^{KO}; *daughterless(da)-GAL4>UAS-THADA*) restores *THADA* expression. Note that the hyperphagia phenotype (D) is more variable than the obesity phenotype (C) since the

hyperphagia phenotype in some genetic backgrounds (e.g. *THADA*^{KO}; *da-GAL4*) is not strong. (n=3x9 adults females per genotype)

(E) *THADA*^{KO} females, but not males, have elevated glycogen levels. Total body glycogen levels in adult flies, normalized to total body protein. (n=3x8 adult flies per genotype)

(F-G) *THADA*^{KO} animals do not have significantly altered circulating sugar levels. Circulating glucose (F) and trehalose (G) were determined from hemolymph isolated from wandering third instar female larvae. (n=3x8)

Error bars: Std. dev. *p<0.05, **p<0.01, ***p<0.001, “n.s.” p=0.47(F) or 0.13(G) by student t-test.

Figure S3: THADA knockout flies do not have ectopic lipid deposition. Related to Figure 1.

(A) Oil Red staining for neutral lipids reveals little to no ectopic lipid deposition in THADA^{KO} guts, a typical organ for ectopic lipid deposition in Drosophila.

(B) Adh-GAL4 and mir278-GAL4 are predominantly expressed in the adult fat body. Expression of the two GAL4 drivers revealed by crossing to UAS-GFP and imaging GFP fluorescence. Images of all tissues for one genotype were taken with the same laser and confocal settings.

Figure S4: Specificity controls for anti-THADA antibody and lack of strong THADA signal in mitochondrial preparations. Related to Figure 2.

(A-B) Control immunostainings of wing discs with α-dTHADA antibody show that it specifically detects THADA protein. Wing imaginal discs expressing either *UAS-THADA* to overexpress *THADA* (A) or *THADA RNAi* to reduce expression of endogenous *THADA* (B) in a central stripe of the wing disc using the *patched-Gal4* driver.

(C) Only very low levels of THADA protein are found in preparations of crude mitochondria from flies. Porin serves as a control for mitochondria, Lamin C for nuclei, and Glyoxalase I for cytosol.

Figure S5: Support to main Figure 4.

(A) Sensitivity to cold exposure of *THADA^{KO}* flies is rescued by ubiquitous expression of *THADA* from a UAS transgene. Adult flies were kept for 5 hours at 4°C to immobilize them. Recovery at room temperature was monitored as the ability to resume climbing. Parental genotypes containing the *THADA* mutation and either the ubiquitous *daughterless-GAL4* (*DaG4*) or *UAS-THADA* do not express THADA, whereas the combination (*THADA^{KO}; DaGAL4>UAS-THADA*) restores *THADA* expression. Sensitivity curve of the rescued animals is significantly different compared to *THADA^{KO};DaG4 animals* (Log-rank test p-score <0.0001). (n=3x18 per genotype)

(B) Ubiquitous expression of SERCA-RNAi in control flies does not reduce their total body triglyceride levels. Total body triglyceride levels normalized to

total body protein for adult female flies with a mild, ubiquitous SERCA knockdown using *daughterless-GAL4* (*daG4>SercaRi*), compared to the parental control genotypes carrying either the GAL4 only or the *UAS-SERCA-RNAi* only. (Error bars: Std. Dev. n=3 x 8).

Supplemental Tables

Table S1: THADA interacting proteins. (Related to Figure 2).

Available as an excel sheet for download from the website.

Table S2: Cloning and gene expression knockdown Oligonucleotides. (Related to STAR Methods)

Oligo Name	Purpose	Sequence
GCA001	Clone upstream flank for THADA knockout #1 construct (AvrII)	ccggcctaggGGGCGGTTGTCGAGGATGTG
GCA004	Clone upstream flank for THADA knockout #1 construct (AscI)	ccggggcgcgccGCATTGTGCGCAGCTATCAT
GCA005	Clone downstream flank for THADA knockout #1 construct (NheI)	ccgggctagcGCCGCCAGGTCATTGAGGAG
GCA008	Clone downstream flank for THADA knockout #1 construct (NotI)	ccgggcggccgcGCTGTGCGCATGGAGAGATC
OAM099	clone downstream flank	TATGGGCGCACTGGGTTAGCAGA

	for THADA knockout #2	
OAM100	clone downstream flank for THADA knockout #2	actagtTTCAGATGGGCGAAGAAATCGAT
GCA360	dsRNA1 against THADA CG15618	ggcctaatacgactcactatagggaggATCCGCAATCCTGACAGAAC
GCA 361	dsRNA1 against THADA CG15618	ggcctaatacgactcactatagggaggGTTGGCGGACACGAAGAC
GCA362	dsRNA2 against THADA CG15618	ggcctaatacgactcactatagggaggGGGCCAGGAACTGTATGA
GCA363	dsRNA2 against THADA CG15618	ggcctaatacgactcactatagggaggAGCCTGATGGCGAAGATA
OAM155	part 1 hThada upper	agatctCCGACGTGCACGAGTGACTACT
OAM156	part 1 hThada lower	ACTCCTGGAGACTGGCTGTTAA
OAM157	part 2 hThada upper	GGGAGAACCTCGTGTCTGATGC
OAM158	part 2 hThada lower	GCACTGCCCAGACCGGTGATGT
OAM159	part 3 hThada upper	AGATACGCGCCTGGGAGAAAAT
OAM160	part 3 hThada lower	gcggccgcCCCCATCCCAATCCCCCAGATT
THADA siRNA pool	Dharmacon	Cat#D-032022
