# Supplementary material for: THADA Regulates the Organismal Balance between Energy Storage and Heat Production
Source: Dev Cell. 2017 Apr 10;41(1):72–81.e6. doi: 10.1016/j.devcel.2017.03.016 (PMC5392496; doi:10.1016/j.devcel.2017.03.016)
Supplement: Document S2. Article plus Supplemental Information [file mmc3.pdf]

Developmental Cell

Article

THADA Regulates the Organismal Balance between Energy Storage and Heat Production

Highlights

- *Drosophila* knockouts of the conserved gene *THADA* are obese and hyperphagic
- *THADA* knockouts produce less heat and are cold sensitive
- THADA binds SERCA and uncouples its ATP hydrolysis from Ca^{2+} pumping
- Reducing SERCA activity rescues the *THADA* loss-of-function phenotypes

Authors

Alexandra Moraru,
Gulcin Cakan-Akdogan,
Katrin Strassburger, ...,
Bart P. Braeckman, Markus Ralser,
Aurelio A. Teleman

Correspondence

a.teleman@dkfz.de

In Brief

One theory for variable human susceptibility to obesity is altered metabolic rates due to adaptation to warmer climates. Moraru et al. examine the function of *THADA*, a positively selected gene in human evolution associated with type 2 diabetes, in *Drosophila* and show that THADA modulates, via calcium signaling, energy storage and thermogenesis balance.

CrossMark

Moraru et al., 2017, Developmental Cell *41*, 72–81
April 10, 2017 © 2017 The Francis Crick Institute. Published by Elsevier Inc.
http://dx.doi.org/10.1016/j.devcel.2017.03.016

CellPress

Developmental Cell
Article

THADA Regulates the Organismal Balance between Energy Storage and Heat Production

Alexandra Moraru,[1,5] Gulcin Cakan-Akdogan,[1,5,6] Katrin Strassburger,[1] Matilda Males,[1] Sandra Mueller,[1] Markus Jabs,[1] Michael Muelleder,[2,4] Martin Frejno,[1] Bart P. Braeckman,[3] Markus Ralser,[2,4] and Aurelio A. Teleman[1,7,*]
[1]German Cancer Research Center (DKFZ), 69120 Heidelberg, Germany
[2]Department of Biochemistry and Cambridge Systems Biology Centre, University of Cambridge, 80 Tennis Court Road, Cambridge CB2 1GA, UK
[3]Department of Biology, Ghent University, 9000 Ghent, Belgium
[4]Molecular Biology of Metabolism Laboratory, The Francis Crick Institute, 1 Midland Road, London NW1 1AT, UK
[5]Co-first author
[6]Present address: Izmir Biomedicine and Genome Center (iBG-izmir), Dokuz Eylül University, 35340 Izmir, Turkey
[7]Lead Contact
*Correspondence: a.teleman@dkfz.de
http://dx.doi.org/10.1016/j.devcel.2017.03.016

SUMMARY

Human susceptibility to obesity is mainly genetic, yet the underlying evolutionary drivers causing variation from person to person are not clear. One theory rationalizes that populations that have adapted to warmer climates have reduced their metabolic rates, thereby increasing their propensity to store energy. We uncover here the function of a gene that supports this theory. *THADA* is one of the genes most strongly selected during evolution as humans settled in different climates. We report here that *THADA* knockout flies are obese, hyperphagic, have reduced energy production, and are sensitive to the cold. THADA binds the sarco/ER Ca^{2+} ATPase (SERCA) and acts on it as an uncoupler. Reducing SERCA activity in *THADA* mutant flies rescues their obesity, pinpointing SERCA as a key effector of THADA function. In sum, this identifies THADA as a regulator of the balance between energy consumption and energy storage, which was selected during human evolution.

INTRODUCTION

Obesity has reached pandemic proportions, with 13% of adults worldwide being obese (World Health Organization, 2015). Although the modern diet triggers this phenotype, 60%–70% of an individual's susceptibility to obesity is genetic (Aguilera et al., 2013; Nan et al., 2012; Naukkarinen et al., 2012). The underlying evolutionary drivers that cause susceptibility to vary from person to person are not clear. Since obesity is most prevalent in populations that have adapted to warm climates, an emerging theory proposes that populations in warm climates evolved low metabolic rates to reduce heat production, making them prone to obesity. In contrast, populations in cold climates evolved high energy consumption for thermogenesis, making them more resistant to obesity (Sellayah et al., 2014). This theory predicts the existence of genes that have been selected in the human population by climate adaptation which regulate the balance between heat production and energy storage.

The gene *Thyroid Adenoma Associated* (*THADA*) has played an important role in human evolution. Comparison of the Neanderthal genome with the genomes of current humans reveals that SNPs in *THADA* were the most strongly positively selected SNPs genome-wide in the evolution of modern humans (Green et al., 2010). Furthermore, as hominins left Africa circa 70,000 years ago, they adapted to colder climates. Genome-wide association studies (GWAS) identified *THADA* as one of the top genes that was evolutionarily selected in response to cold adaptation (Cardona et al., 2014), suggesting a link between *THADA* and energy metabolism. *THADA* was also identified as one of the top risk loci for type 2 diabetes by GWAS (Zeggini et al., 2008). Although follow-up studies could not confirm an association between *THADA* SNPs and various aspects of insulin release or insulin sensitivity (Grarup et al., 2008; Sanghera et al., 2009; Schleinitz et al., 2010; Staiger et al., 2008; Stancakova et al., 2009), some studies did find an association between *THADA* and pancreatic β-cell response (Simonis-Bik et al., 2010) or marginal evidence for an association with body mass index (Gupta et al., 2013). In sum, *THADA* has been connected to both metabolism and adaptation to climate. Nonetheless, to our knowledge nothing is known about the function of THADA in animal biology, at the physiological or the molecular level. Animals lacking THADA function have not yet been described. An analysis of the amino acid sequence of THADA provides little or no hints regarding its molecular function.

To study the function of THADA, we generated *THADA* knockout flies. *THADA* knockout animals are obese and produce less heat than controls, making them sensitive to the cold. We find that THADA binds the sarco/ER Ca^{2+} ATPase (SERCA) and regulates organismal metabolism via calcium signaling. In addition to unveiling the physiological role and molecular function of this medically relevant gene, our results also show that one gene that has been strongly selected during human evolution in response to environmental temperature plays a functional role in regulating the balance between heat production and energy storage, affecting the propensity to become obese.

 © 2017 The Francis Crick Institute. Published by Elsevier Inc.
This is an open access article under the CC BY license (http://creativecommons.org/licenses/by/4.0/).

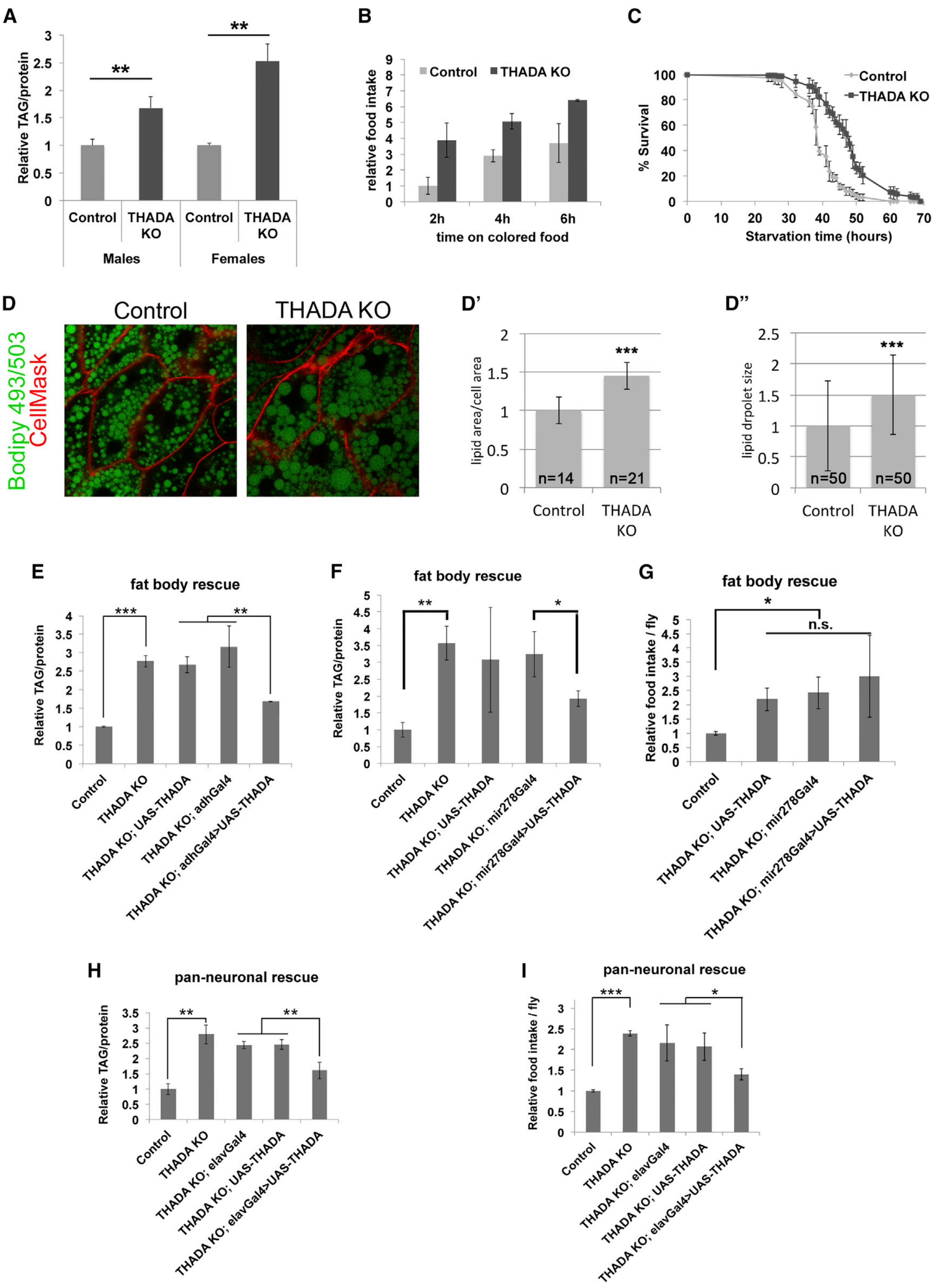

Figure 1. THADA Knockout Flies Are Obese

(A) *THADA*^{KO1} animals have strongly elevated total body triglyceride levels, normalized to total body protein content (n = 3 × 8 3-day-old adults).

(B) *THADA*^{KO} animals are hyperphagic. Relative food intake in *THADA*^{KO} adult female flies, quantified by transferring flies from normal food to food containing Acid Blue 9 for indicated times, followed by fly washing, homogenization, and measurement of OD625 (n = 3 × 9).

(C) *THADA*^{KO} animals are resistant to starvation. Adult female flies were transferred from normal food to 1% agarose/PBS and survivorship was quantified over time (n = 4 × 50).

(legend continued on next page)

RESULTS

THADA Mutant *Drosophila* Have Metabolic Defects

A BLAST search of the *Drosophila* proteome with the protein sequence of human THADA (NP_071348.3) identifies CG15618 as the top hit with an E value of 10^{-73}. Conversely, a BLAST search of the human proteome using the protein sequence of CG15618 identifies hTHADA as the top hit, thereby establishing an orthologous relationship between hTHADA and CG15618 (Figure S1A). Hence, we refer to CG15618 as *Drosophila* THADA. To study THADA function, we generated *THADA* knockout flies by homologous recombination to generate two different loss-of-function alleles (Figure S1B). Allele KO1 deletes a 1,024-bp genomic region comprising the fourth exon and part of the fifth exon of *THADA*, causing the rest of the *THADA* open reading frame to be out of frame, whereas allele KO2 is circa 1 kb larger and also deletes the transcription start site of *THADA* (Figure S1B). Both alleles lead to loss of THADA protein (Figure S1C) and have identical phenotypes, so the following data will refer to the KO1 allele (henceforth $THADA^{KO}$) unless otherwise specified. $THADA^{KO}$ are viable (97% survive embryogenesis; survival to adulthood is shown in Figure S1D) and have normal patterning (not shown), developmental timing (Figures S1E and S1E′), adult wing size (a sensitive proxy for body size, Figures S1F and S1F′), cell size (Figures S1F and S1F′), and life span (Figures S1G and S1G′). Since *THADA* has been associated with type 2 diabetes (Zeggini et al., 2008), we asked whether THADA regulates organismal metabolism. Indeed, both $THADA^{KO1}$ and $THADA^{KO2}$ flies have strongly elevated triglyceride levels (Figures 1A and S2A). $THADA^{KO}$ are also hyperphagic, measured by quantifying ingestion of colored food (Figures 1B and S2B). The obese and hyperphagic phenotypes are specific for *THADA* loss of function, since they can be rescued by ubiquitous expression of *THADA* from a transgene (Figures S2C and S2D). Compared with the increased adiposity of $THADA^{KO}$ animals, the increased-feeding phenotype is less robust and varies depending on genetic background (e.g., $THADA^{KO}$, *da-GAL4* flies do not eat as much as $THADA^{KO}$, Figure S2D). Hence, we focus here on the adiposity. Like mammals, *Drosophila* store energy both as triglycerides and as glycogen. Flies with elevated triglycerides frequently have elevated glycogen. We quantified total body glycogen and found it to be significantly elevated in $THADA^{KO}$ females but not males (Figure S2E). Another phenotype that correlates with elevated nutrient stores in *Drosophila* is starvation resistance; when subjected to starvation, flies die in coincidence with depletion of their energy stores (Teleman et al., 2005). Consistently, $THADA^{KO}$ animals were resistant to starvation compared with controls (Figure 1C). In contrast, levels of circulating sugars, glucose and trehalose, were not significantly altered in $THADA^{KO}$ animals (Figures S2F and S2G).

To study the $THADA^{KO}$ obesity in more detail, we first asked whether the excess lipids accumulate ectopically in the body. Oil red O staining of tissues, however, did not reveal noticeably elevated levels of neutral lipids in ectopic locations such as the gut (Figure S3A). Instead, the fat body—the *Drosophila* equivalent of adipose tissue and liver combined—in $THADA^{KO}$ animals contains larger lipid droplets and more total lipid per cell compared with controls (Figures 1D–1D″), indicating the obesity is mainly due to elevated lipid storage in its normal location, the fat body. To test which tissues contribute toward the increased adiposity of $THADA^{KO}$ animals, we performed rescue experiments re-introducing *THADA* expression in specific tissues. Expression of *THADA* in the fat body using two fat body drivers, adh-GAL4 and mir278-GAL4 (see Figure S3B for expression patterns), partially rescued $THADA^{KO}$ obesity (Figures 1E and 1F), as did expression of *THADA* in all neurons (Figure 1H), indicating that defects in both the fat body and the nervous system contribute to the fat phenotype. Neuronal expression of *THADA*, but not fat body expression of *THADA*, also partially rescued the increased food intake (Figures 1G and 1I). In sum, the obese phenotype of $THADA^{KO}$ is partly due to an autonomous defect in the fat body and partly due to a defect in the nervous system, likely via feeding.

In sum, $THADA^{KO}$ animals are viable and have normal size and patterning, but have elevated triglyceride stores, due to defects in both the fat body and the nervous system. This identifies THADA as a regulator of organismal metabolism.

THADA Localizes to the ER

To understand the molecular function of THADA, we first studied its subcellular localization. We confirmed that our α-THADA antibody specifically recognizes THADA protein in tissue stainings, via control experiments overexpressing or knocking down endogenous THADA in wing discs (Figures S4A and S4B), as it does on immunoblots (Figure S1C). We then compared the subcellular localization of THADA with GFP markers for various organelles, and found that THADA localizes to the ER. In muscle, THADA and the ER marker GFP-KDEL (Yogev et al., 2010) co-localize perfectly (Figure 2A, top row). In the fat body, GFP-KDEL localization is more complex because it marks both ER and Golgi (the KDEL sequence causes retrograde transport of cargo from the Golgi to the ER). In the fat body the Golgi network

(D–D″) Fat body of $THADA^{KO}$ animals stained for neutral lipids with Bodipy 493 (D) reveals that they have more lipid per cell (D′) and larger lipid droplets (D″) compared with controls.

(E and F) $THADA^{KO}$ obesity is partially rescued by re-expressing *THADA* from a transgene in fat body using the bipartite GAL4/UAS system with two different fat body-specific drivers, Adh-GAL4 (E) or mir278-GAL4 (F). Total body triglycerides normalized to total body protein. Parental genotypes containing the $THADA^{KO}$ mutation and either a *GAL4* driver or the *UAS-THADA* transgene, which individually do not cause *THADA* expression, have elevated triglyceride levels. Combining the *GAL4* driver and *UAS-THADA* leads to re-expression of *THADA* specifically in fat body, and partial rescue of the obesity.

(G) $THADA^{KO}$ hyperphagia is not rescued by re-expressing *THADA* in the fat body using mir278-GAL4. Fly genotypes as in (F). Flies were kept on food containing Acid Blue 9 for 4 hr and further processed as in (B) (n = 3 × 9 flies).

(H) $THADA^{KO}$ obesity is partially rescued by re-expressing *THADA* from a transgene in neurons. Expression in all neurons is achieved using the *elav-GAL4* driver (n = 3 × 9 adult females per genotype).

(I) Hyperphagia of $THADA^{KO}$ animals is partially rescued by re-expressing *THADA* specifically in neurons. Relative food intake quantified as in (B) and genotypes as in (H) (n = 3 × 9 adult females).

Error bars denote SD. *p < 0.05, **p < 0.01, ***p < 0.001 by Student's t test. See also Figures S1–S3.

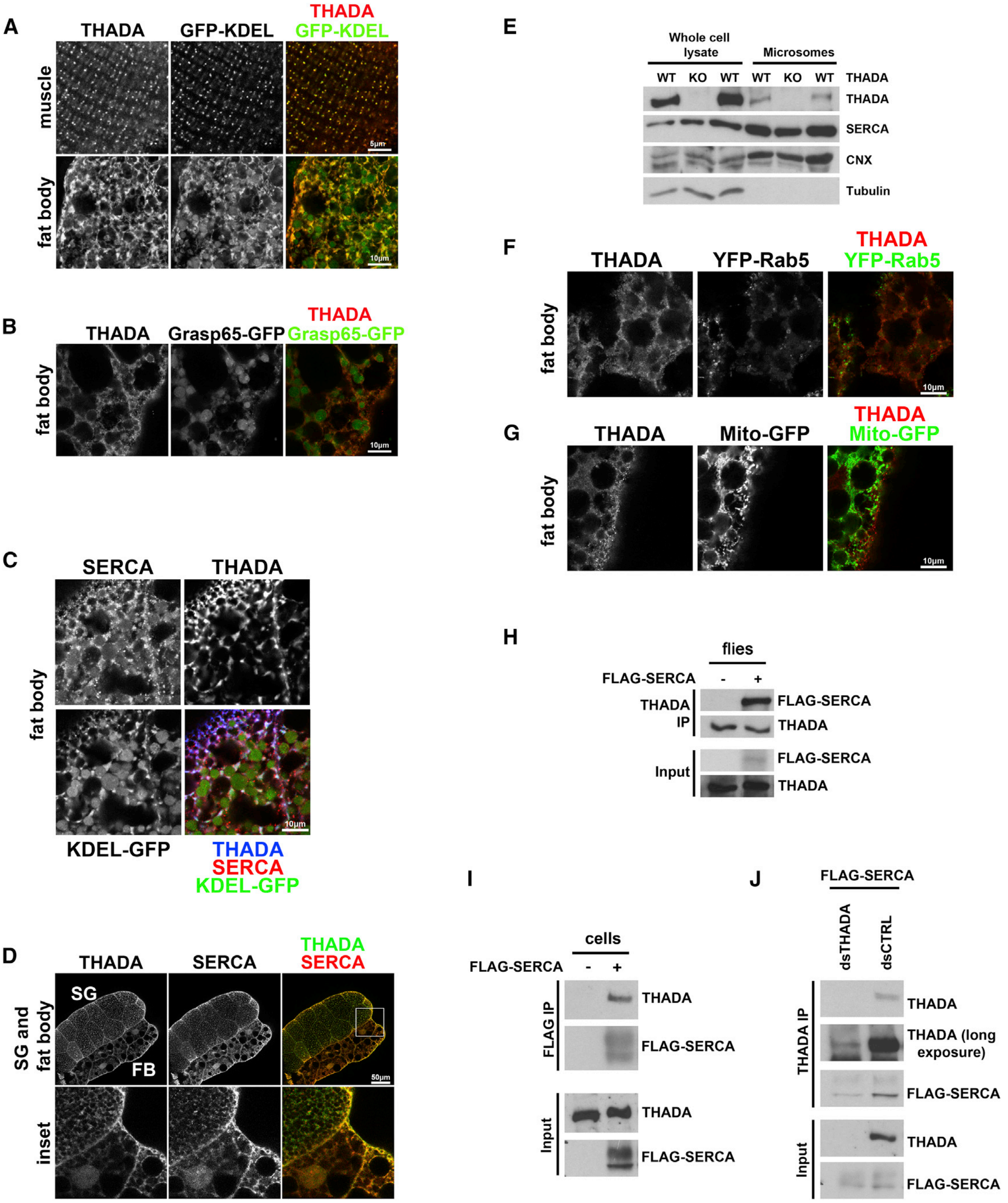

Figure 2. *Drosophila* THADA Localizes to the ER and Interacts Physically with SERCA

(A and B) THADA localizes to the ER and not the Golgi. Larval tissues expressing either GFP-KDEL (A) or the Golgi marker Grasp65-GFP (B) were stained to detect THADA. In muscle GFP-KDEL marks the ER, whereas in fat body GFP-KDEL marks both the ER (stronger reticulate pattern) and the Golgi (weaker round vesicles).

(C) GFP-KDEL marks both the ER and the Golgi in fat body. Fat body expressing GFP-KDEL was immunostained to detect endogenous SERCA and THADA. GFP-KDEL marks both the reticulate ER, stained by the ER marker SERCA, and the Golgi which appears as round vesicles. THADA co-localizes with SERCA on the ER.

(D) THADA co-localizes with SERCA. Larval salivary gland (SG) and fat body (FB) were stained to detect endogenous THADA and SERCA. Lower panels show a 4× magnification of the indicated region (Pearson's R = 0.74 for entire FB + SG).

(E) THADA is present in microsomal preparations, purified by ultracentrifugation of larval lysates. Calnexin (CNX) and tubulin serve as positive and negative controls for ER purification, respectively.

(F) THADA does not localize to early endosomes, marked with YFP-Rab5. Fat body from animals expressing YFP-Rab5, immunostaining to detect THADA localization.

(G) THADA does not localize to mitochondria, marked with mito-GFP. Fat body from animals expressing mito-GFP, immunostaining to detect THADA localization.

(legend continued on next page)

has a round appearance, as seen using the Golgi-specific marker Grasp65-GFP (Figure 2B), which does not co-localize with THADA (Figure 2B). In the fat body GFP-KDEL marks these round Golgi structures that do not co-localize with THADA, as well as the more reticulate ER network, which stains positive for the ER marker SERCA (Figure 2C) and co-localizes with THADA (Figures 2C and 2A, bottom panel). Indeed, SERCA, which is widely used as an ER marker, co-localizes with THADA in all tissues tested such as the fat body and salivary gland (Figure 2D). Consistent with this, we detected THADA in microsomal preparations (Figure 2E). Since THADA has no obvious signal sequence, this suggests it localizes to the cytosolic side of the ER membrane. In contrast, we detected no co-localization with other organelle markers such as YFP-Rab5 (early endosomes) or Mito-GFP (mitochondria) (Figures 2F and 2G) and only very low levels of THADA in purified mitochondria (Figure S4C). In sum, THADA mainly localizes to the ER, co-localizing with SERCA.

THADA Interacts with SERCA and Regulates Its Activity

Since the amino acid sequence of THADA yields no hints regarding its molecular function, we aimed to identify proteins interacting with THADA. We immunoprecipitated endogenous THADA from fly lysates and used mass spectrometry to identify co-precipitating proteins. An equivalent anti-THADA immunoprecipitate from *THADA*^{KO} flies served as a negative control. This identified 228 proteins interacting with THADA (Table S1). From this list of interacting proteins we focused on SERCA, because it was the only protein that we could confirm interacts with THADA in a consistent manner by co-immunoprecipitation experiments, and because SERCA co-localizes with THADA. SERCA is an ATP-dependent calcium pump that transports calcium ions from the cytosol into the ER, and calcium signaling has recently been described to regulate organismal metabolism (Baumbach et al., 2014; Bi et al., 2014; Subramanian et al., 2013a). THADA and SERCA co-immunoprecipitated from both fly and Kc167 cell lysates (Figures 2H–2J).

SERCA and calcium signaling have recently been found to regulate metabolism in *Drosophila* and mice (Bal et al., 2012; Baumbach et al., 2014; Bi et al., 2014; Subramanian et al., 2013b; Ye et al., 2011). *Drosophila* with hypomorphic mutations in the inositol 1,4,5-trisphosphate receptor (IP_3R), which opposes SERCA function by releasing calcium from the ER into the cytosol, have the same phenotypes as the ones we observe in *THADA* mutants: they are obese, starvation resistant, and hyperphagic, due to functions of IP_3R in both fat body and the CNS (Subramanian et al., 2013a, 2013b). This prompted us to study calcium signaling and SERCA in *THADA* mutants.

SERCA affects metabolism via two mechanisms. Firstly, SERCA uses the energy derived from ATP hydrolysis in part to translocate calcium from the cytosol into the ER, and in part to produce heat via a futile cycle (Reis et al., 2001). The importance of this futile ATP hydrolysis for non-shivering thermogenesis is shown by the regulator sarcolipin, which uncouples SERCA ATP hydrolysis from calcium pumping, causing SERCA to produce more heat per molecule of ATP consumed (Bal et al., 2012). In the absence of sarcolipin, animals have reduced thermogenesis and are sensitive to the cold (Bal et al., 2012). Secondly, in addition to an effect on thermogenesis, loss of an SERCA uncoupling protein leads to more efficient calcium pumping by SERCA, thereby elevating ER calcium levels. Elevated ER calcium levels cause obesity and hyperphagia (Baumbach et al., 2014; Subramanian et al., 2013a, 2013b). We asked whether THADA affects SERCA activity. An SERCA activity assay that quantifies Ca^{2+}-dependent ATP hydrolysis (Gehrig et al., 2012) revealed that loss of THADA leads to significantly elevated calcium-dependent SERCA activity both in flies (Figure 3A) and in cells in culture (Figure 3B), indicating that it is a cell-autonomous effect. We next analyzed calcium levels in the cells, whereby we were unable to detect any significant differences in cytosolic calcium levels in *THADA* knockdown cells compared with controls (Figures 3C and 3C′). We then quantified ER calcium levels using two independent methods that measure calcium release from the ER into the cytosol. Cytosolic calcium levels increased more strongly in *THADA* knockdown cells compared with controls, either upon calcium release from the ER with ionomycin (Figure 3D) or upon acutely blocking calcium re-uptake into the ER with thapsigargin (Figure 3E), indicating *THADA* knockdown cells have elevated ER calcium. Together with the SERCA activity assay, these data indicate improved pumping of calcium into the ER in *THADA* mutants, suggesting that THADA acts either as an SERCA inhibitor or SERCA uncoupler.

Loss of either an SERCA uncoupler or an SERCA inhibitor leads to elevated calcium pumping (Figure 4A). However, SERCA uncouplers and SERCA inhibitors have opposite effects on heat production by SERCA. Loss of an SERCA uncoupling protein results in the phenotypes observed in sarcolipin mutant animals, which is reduced heat production and, consequently, cold sensitivity (Figure 4A) (Bal et al., 2012). In contrast, loss of an SERCA inhibitor would lead to increased SERCA-dependent thermogenesis (Figure 4A). To distinguish between these two possibilities, we exposed *THADA*^{KO} flies to 4°C for 3 hr, which causes them to become paralyzed, and then monitored their recovery at room temperature. Interestingly, *THADA*^{KO} flies were significantly impaired in their ability to wake up from cold treatment compared with controls (Figures 4B and S5A), suggesting that they might have impaired heat production. Indeed, quantification of heat production by microcalorimetry revealed that *THADA*^{KO} flies produce mildly but consistently and significantly reduced heat compared with controls (Figure 4C). Together, these data indicate THADA acts as an SERCA uncoupling protein, similar to sarcolipin.

(H) FLAG-SERCA interacts with endogenous THADA. Immunoprecipitation of endogenous THADA from lysates of transgenic adult flies expressing FLAG-SERCA.

(I) FLAG-SERCA interacts with endogenous THADA in Kc167 cells. FLAG-SERCA immunoprecipitates from transfected Kc167 cells were immunoblotted with indicated antibodies.

(J) Endogenous THADA co-immunoprecipitates FLAG-SERCA from Kc167 lysates. Lysates from THADA knockdown cells serve as a negative control.

See also Figure S4.

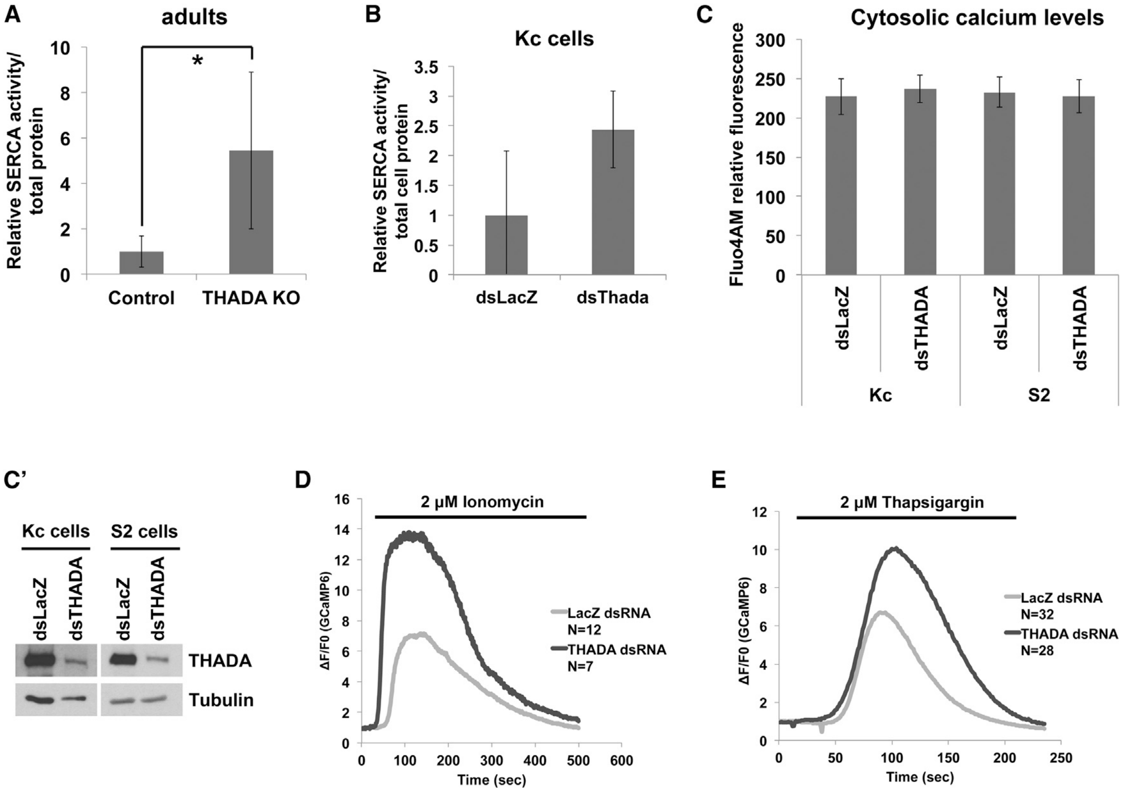

Figure 3. *THADA* Mutants Have Elevated SERCA Activity

(A) Relative SERCA activity in lysates of control and $THADA^{KO}$ animals. Activity measured as Ca^{2+}-dependent ATP hydrolysis (Gehrig et al., 2012), normalized to total protein content. Error bars denote SD. *$p < 0.05$ by Student's t test. n = 4 × 15 adult females.

(B) Relative SERCA activity in lysates of control (*LacZ*) knockdown Kc167 or *THADA* knockdown Kc167 cells. Activity was measured as Ca^{2+}-dependent ATP hydrolysis (Gehrig et al., 2012), normalized to total protein content (error bars denote SD; n = 3).

(C and C′) Cytosolic calcium levels are not detectably altered in $THADA^{KO}$ cells compared with control cells. (C) Cytosolic calcium levels quantified as Fluo4AM fluorescent intensity per cell (error bars denote SD; n = 430–1,330 cells). (C′) Control for *THADA* knockdown efficiency detected by immunoblotting.

(D and E) *THADA* knockdown causes elevated ER calcium levels, observed as a larger increase in cytosolic calcium when calcium is released from the ER. Fluorescence intensity change ($\Delta F/F_0$) in *Drosophila* $S2R^+$ cells (D) and Kc167 cells (E) expressing the cytosolic Ca^{2+} sensor GCaMP6 and treated with either 2 μM ionomycin (D) or 2 μM thapsigargin (E). In both cases, culture medium was supplemented with 10 mM EGTA.

$THADA^{KO}$ Obesity Is Rescued by Reducing SERCA Activity

Since elevated ER calcium leads to increased adiposity (Baumbach et al., 2014; Bi et al., 2014; Subramanian et al., 2013a, 2013b), this could explain the obesity we observe in *THADA* mutants. To test this, we asked whether we could rescue the obesity of $THADA^{KO}$ flies by reducing SERCA expression. Strong, ubiquitous knockdown of SERCA using the *Tubulin-GAL4* driver is lethal (not shown), whereas a mild ubiquitous SERCA knockdown using *daughterless-GAL4* in a wild-type background leads to viable adults with no significant changes in total body triglyceride levels (*DaG4>SERCA-RNAi*, Figures 4D and S5B). This mild, ubiquitous knockdown of SERCA completely reversed the obesity of *THADA* mutants (Figure 4D), indicating that the obesity is indeed a result of improved ER calcium pumping in these animals. While SERCA pumps calcium from the cytosol into the ER, IP_3R performs the reverse, releasing calcium from the ER into the cytosol. Consistent with this, the obesity of *THADA* mutants was also partially rescued by overexpression of IP_3R (Figure 4E). Since THADA modulates thermogenesis, and *THADA* mutant flies are cold sensitive, we asked whether THADA protein or mRNA levels are modulated by exposing flies to different temperatures, but this was not the case (Figures 4F and 4G).

THADA Molecular Function Is Conserved from Flies to Humans

We asked whether the molecular function of THADA is conserved between flies and humans. We first tested whether human THADA can replace *Drosophila* THADA in the fly. Expression of human *THADA* in $THADA^{KO}$ flies rescued their obese phenotype (Figure 5A), indicating that human THADA can compensate for loss of fly THADA, and hence the molecular functions of THADA are likely conserved. We then tested whether loss of THADA in human cells leads to elevated ER calcium, as it does in fly cells. Indeed, using the ER calcium release assay, we found that *THADA* knockdown HeLa cells have elevated ER calcium levels compared with controls (Figure 5B).

DISCUSSION

We report here the physiological and molecular function of THADA in animals. We find that *THADA* mutants are obese,

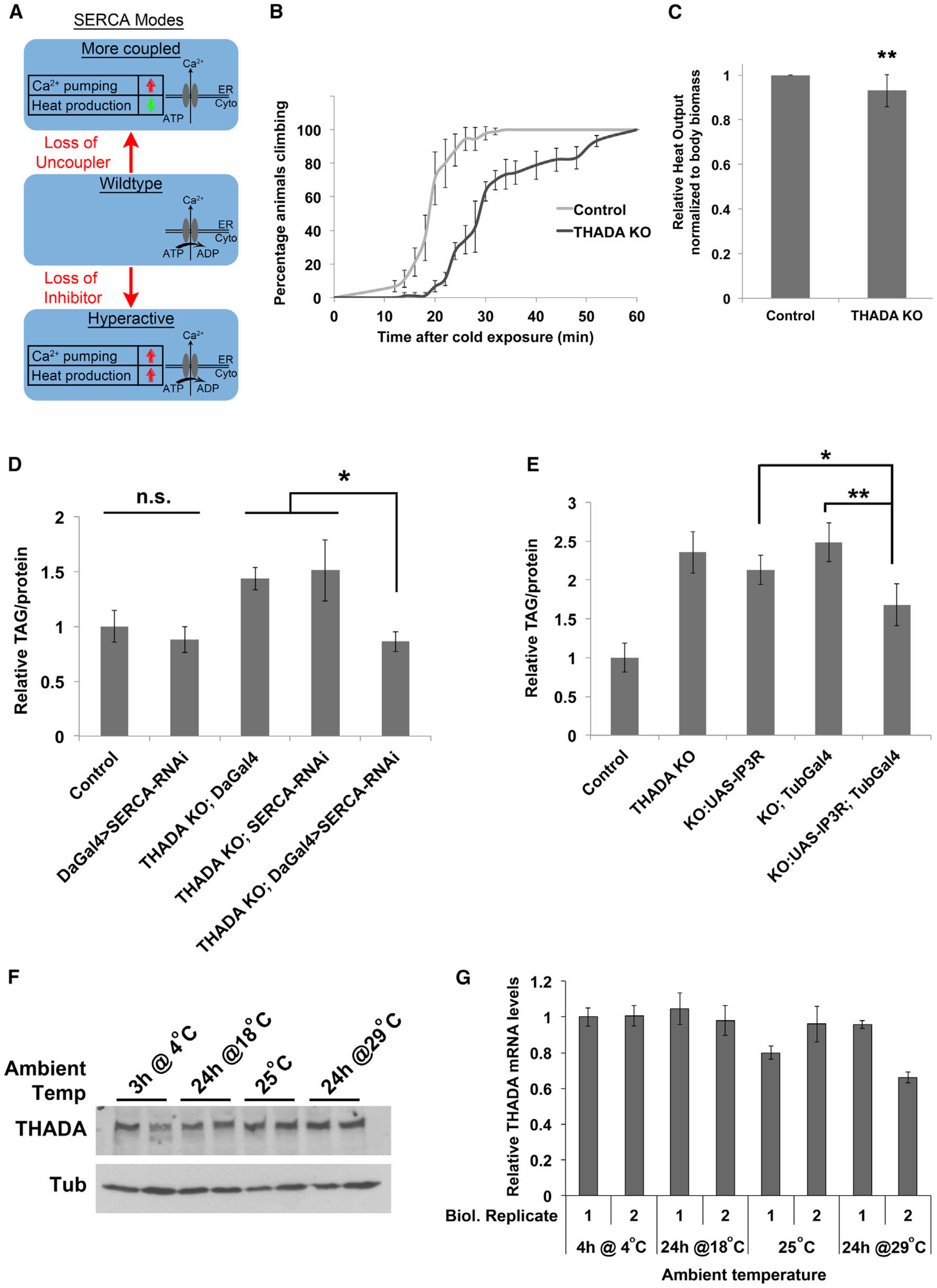

Figure 4. *THADA* Acts as a SERCA Uncoupling Protein

(A) Schematic outlining SERCA activity when it is coupled, uncoupled, or hyperactive.

(B) $THADA^{KO}$ flies are hypersensitive to the cold. Adult flies were kept for 3 hr at 4°C to immobilize them. Recovery at room temperature was monitored as the ability to resume climbing (error bars denote SD; n = 3 × 30 adult flies).

(C) $THADA^{KO}$ flies generate less heat than control flies. Heat production at 25°C quantified by microcalorimetry, and normalized to total body biomass (fly weight). Error bars denote SD. n = 12 × 5 flies. **p < 0.01 (t test).

(legend continued on next page)

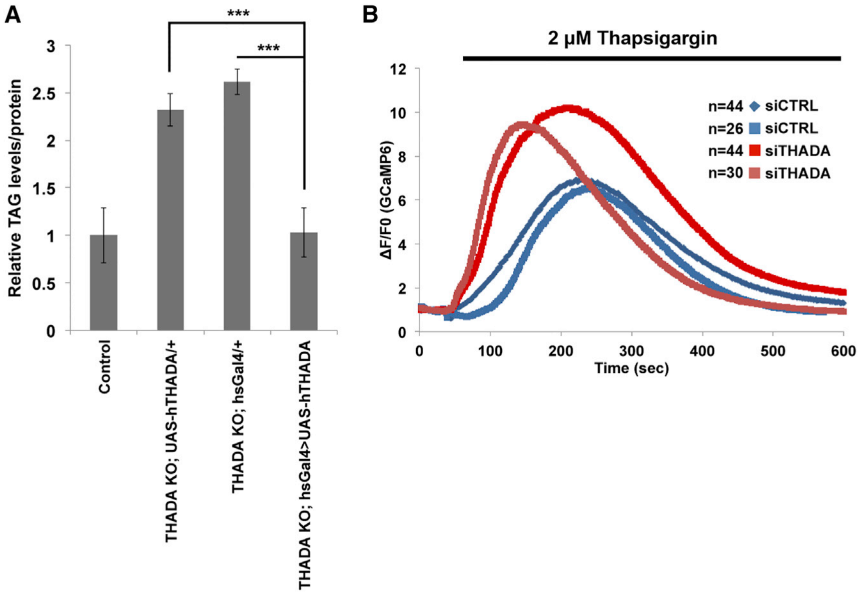

Figure 5. Molecular Functions of dTHADA Are Conserved in hTHADA

(A) Obesity of *THADA*^{KO} flies is rescued by ubiquitous expression of human THADA (hTHADA). Control parental genotypes (*THADA KO*; *UAS-hTHADA* and *THADA KO*; *heat shock-GAL4*) are obese, whereas the combined genotype which expresses human THADA in the THADA knockout background (*THADA KO*; *hsGAL4*>*UAS-hTHADA*) are not. Error bars denote SD. n = 4 × 8 adult female flies. *** $p < 0.001$ (t test).

(B) *THADA* knockdown HeLa cells have elevated ER calcium levels, observed as a larger increase in cytosolic calcium when calcium is released from the ER. Fluorescence intensity change ($\Delta F/F_0$) in cells expressing the cytosolic Ca^{2+} sensor GCaMP6 and treated with 2 μM thapsigargin. Culture medium was supplemented with 2 mM EGTA. Two biological replicates are shown.

See also Figure S5.

sensitive to the cold, and have reduced heat production compared with controls. We find that THADA interacts physically with SERCA and modulates its activity. The combination of improved calcium pumping and cold sensitivity of *THADA* mutants indicates that THADA acts as an SERCA uncoupler, similar to sarcolipin. This interaction between THADA and SERCA appears to be an important part of THADA function, since the obesity phenotype of *THADA* mutants can be rescued by mild *SERCA* knockdown.

Calcium signaling is increasingly coming into the spotlight as an important regulator of organismal metabolism. In a genome-wide in vivo RNAi screen in *Drosophila* to search for genes regulating energy homeostasis, calcium signaling was the most enriched gene ontology category among obesity-regulating genes (Baumbach et al., 2014). Cytosolic calcium levels can alter organismal adiposity by more than 10-fold (from 15% to 250% of control levels) (Baumbach et al., 2014), indicating that it is an important regulator of organismal metabolism. In line with these numbers, *THADA*^{KO} flies have 250% the triglyceride levels of control flies. The phenotypes observed for other regulators of calcium signaling all point in the same general direction that high ER calcium leads to hyperphagia and obesity (Bi et al., 2014; Subramanian et al., 2013a). Likewise, mice heterozygous for a mutation in IP_3R are susceptible to developing glucose intolerance on a high-fat diet (Ye et al., 2011).

The molecular mechanisms by which ER calcium regulates organismal metabolism are not yet fully understood, but this important question will surely be the subject of intense research in the near future. Calcium levels are known to regulate activity of tricarboxylic acid cycle enzymes such as α-ketoglutarate dehydrogenase, isocitrate dehydrogenase, and pyruvate dehydrogenase (Kaufman and Malhotra, 2014), which could explain part of the effect of calcium on metabolism.

We find that *THADA* mutation leads to obesity due to roles of THADA both in the fat body and in neurons. This has also been observed for IP_3R mutants (Subramanian et al., 2013b). Calcium signaling regulates lipid homeostasis directly and cell-autonomously in the fat body, as observed in seipin mutants (Bi et al., 2014) or when Stim expression was modulated specifically in fat tissue (Baumbach et al., 2014). In addition, it regulates feeding via the CNS (Baumbach et al., 2014; Subramanian et al., 2013a). Interestingly, while *THADA* mutant females have elevated glycogen levels, *THADA* mutant males do not. We do not know why this is the case: it could be due to the higher energetic demand in females compared with males, leading to stronger metabolic phenotypes in females, or THADA might regulate glycogen metabolism differently in the two sexes.

GWAS identified *THADA* as one of the top risk loci for type 2 diabetes (Zeggini et al., 2008). The data presented here indicate that THADA regulates lipid metabolism and feeding, suggesting that the association between *THADA* and diabetes may be causal in nature. *THADA* mutant flies develop obesity, but have normal circulating sugar levels under our standard laboratory food conditions. Interestingly, mouse mutants for IP_3R likewise do not become insulin resistant under a regular diet, but do

(D) Reducing SERCA activity rescues the elevated triglyceride phenotype of *THADA*^{KO} flies. Relative triglyceride levels in 6-day-old adult females, normalized to total body protein. Mild ubiquitous SERCA knockdown is achieved by combining a weak ubiquitous GAL4 driver (*daughterless-GAL4*) with an inducible transgene *UAS-SERCA-RNAi*. Error bars denote SD. *$p < 0.05$; n.s., not significant ($p = 0.3$) by Student's t test. n = 3 × 8 adult female flies per genotype.

(E) Increasing IP3R activity rescues the elevated triglyceride phenotype of *THADA*^{KO} flies. Relative triglyceride levels in 6-day-old adult females, normalized to total body protein. Ubiquitous IP3R expression is achieved by combining the ubiquitous *Tubulin-GAL4* driver with an inducible transgene *UAS-IP3R*. Error bars denote SD. *$p < 0.05$, **$p < 0.01$ by Student's t test. n = 3 × 8 adult female flies per genotype.

(F and G) THADA protein (F) or mRNA levels (G) do not change strongly upon exposing flies to different temperatures. All conditions were tested in biological duplicates. Adult female flies were shifted to the indicated temperatures for the indicated times prior to lysis for immunoblot or qRT-PCR analysis.

become insulin resistant on a high-fat diet (Ye et al., 2011). Combined, these data suggest that the primary effect of altered THADA activity and calcium signaling is on lipid metabolism, and that a combination with high-fat feeding may be required to lead to type 2 diabetes over time. This could potentially explain why follow-up association studies did not find links between *THADA* and insulin sensitivity (Grarup et al., 2008; Sanghera et al., 2009; Schleinitz et al., 2010; Staiger et al., 2008; Stancakova et al., 2009) but did find links between *THADA* and adiposity (Gupta et al., 2013).

Insects are ectotherms, meaning that their internal physiological sources of heat are not sufficient to control their body temperature. Nonetheless they do produce heat, and the main sources of heat are either of muscular origin due to movement or shivering, or of biochemical origin from futile cycles that consume ATP with no net work (Loli and Bicudo, 2005). For instance, bumblebees preheat their flight muscles by simultaneously activating phosphofructokinase and fructose 1,6-bisphosphatase, which catalyze opposing enzymatic reactions, leading to the futile hydrolysis of ATP and release of heat (Loli and Bicudo, 2005). *Drosophila* also have mitochondrial uncoupling proteins, which potentially generate a futile metabolic cycle by dissipating the mitochondrial membrane potential (Hanak and Jezek, 2001; Loli and Bicudo, 2005). We propose here that uncoupled hydrolysis of ATP by SERCA could constitute one additional example of such a futile cycle that produces heat. We cannot exclude, however, that *THADA* knockout flies might also have changes in their evaporative heat loss contributing to their reduced thermogenesis. The thermogenic phenotypes in *THADA* knockout flies are relatively mild, perhaps reflecting the ectothermic nature of flies. Hence it will be of interest to study in the future the metabolic parameters of *THADA* knockout mice.

The combination of cold sensitivity and obesity in *THADA* mutant animals is interesting in terms of the evolutionary origins of the current obesity pandemic. The prevalence of obesity is highest in populations that have adapted to warmer climates, suggesting that people in warm climates evolved reduced metabolic rates to prevent overheating, and in combination with our modern diet this reduced metabolic rate leads to obesity (Sellayah et al., 2014). Interestingly, *THADA* is a gene that provides support for this theory. SNPs in *THADA* are among the SNPs genome-wide that have been most strongly selected as humans adapted to climates of different temperatures (Cardona et al., 2014). Indeed, comparison of the Neanderthal genome with the genomes of current humans reveals that SNPs in *THADA* were the most strongly positively selected SNPs genome-wide in the evolution of modern humans (Green et al., 2010). The data we present here show that THADA simultaneously affects sensitivity to cold and obesity. Uncoupled SERCA ATPase activity is a major contributor to non-shivering thermogenesis (Bal et al., 2012). Similar to animals mutant for another SERCA uncoupling protein, sarcolipin (Bal et al., 2012), we find that *THADA* mutants are sensitive to the cold. This provides a possible explanation for why evolution selected for SNPs in *THADA*. In addition, THADA, via SERCA, also regulates lipid homeostasis. THADA thereby provides a genetic and molecular link between climate adaptation and obesity.

STAR★METHODS

Detailed methods are provided in the online version of this paper and include the following:

- KEY RESOURCES TABLE
- CONTACT FOR REAGENT AND RESOURCE SHARING
- EXPERIMENTAL MODEL AND SUBJECT DETAILS
  - Experimental Animals
  - Human Cell Lines
  - *Drosophila* Cell Lines
- METHOD DETAILS
  - Generation of THADA Knockout and Rescue Flies
  - Fly Rearing Conditions for Metabolic Analyses
  - Food Intake, TAG and Glycogen Measurements
  - Pupation Curves
  - Starvation Resistance
  - Wing Size and Cell Size
  - Lifespan
  - Circulating Trehalose & Glucose Measurements
  - Serca Activity Assay
  - Microcalorimetry
  - Cold Sensitivity Recovery Assay
  - Bodipy Staining
  - Oil Red Staining
  - Antibody Production
  - Other Antibodies
  - Immunostainings
  - Cell Culture and dsRNA Treatments
  - Plasmid Transfection
  - GCaMP6 Fluorescent Assays
  - Fluo4AM Staining
  - Immunoprecipitation
  - Microsome Isolations
  - Crude Mitochondrial Preparations
  - Human THADA cDNA Cloning
- QUANTIFICATION AND STATISTICAL ANALYSIS
  - Quantifications
  - Statistical Analyses

SUPPLEMENTAL INFORMATION

Supplemental Information includes five figures and two tables and can be found with this article online at http://dx.doi.org/10.1016/j.devcel.2017.03.016.

AUTHOR CONTRIBUTIONS

A.M., G.C.-K., K.S., M.M., S.M., M.J., M.M., M.F., and A.A.T. conducted experiments and, along with B.P.B. and M.R., designed the experiments and/or analyzed results. A.M., K.S., and A.A.T. wrote the paper.

ACKNOWLEDGMENTS

We thank Katrine Weischenfeldt and Marilena Lutz for technical assistance and Martina Schnölzer and the DKFZ Proteomics Core Facility for mass spectrometry analyses. This work was partly funded by a Deutsche Krebshilfe grant (#109118) and by a Deutsche Forschungsgemeinschaft Sonderforschungsbereich SFB1118 grant to A.T. M.R. was supported by the Francis Crick Institute, which receives its core funding from Cancer Research UK (FC001134), the UK Medical Research Council (FC001134), and the Wellcome Trust (FC001134).

Received: October 7, 2016
Revised: February 22, 2017
Accepted: March 15, 2017
Published: April 10, 2017

REFERENCES

Aguilera, C.M., Olza, J., and Gil, A. (2013). Genetic susceptibility to obesity and metabolic syndrome in childhood. Nutr. Hosp. *28* (*Suppl 5*), 44–55.

Bal, N.C., Maurya, S.K., Sopariwala, D.H., Sahoo, S.K., Gupta, S.C., Shaikh, S.A., Pant, M., Rowland, L.A., Bombardier, E., Goonasekera, S.A., et al. (2012). Sarcolipin is a newly identified regulator of muscle-based thermogenesis in mammals. Nat. Med. *18*, 1575–1579.

Baumbach, J., Hummel, P., Bickmeyer, I., Kowalczyk, K.M., Frank, M., Knorr, K., Hildebrandt, A., Riedel, D., Jackle, H., and Kuhnlein, R.P. (2014). A *Drosophila* in vivo screen identifies store-operated calcium entry as a key regulator of adiposity. Cell Metab. *19*, 331–343.

Bi, J., Wang, W., Liu, Z., Huang, X., Jiang, Q., Liu, G., Wang, Y., and Huang, X. (2014). Seipin promotes adipose tissue fat storage through the ER Ca(2)(+)-ATPase SERCA. Cell Metab. *19*, 861–871.

Cardona, A., Pagani, L., Antao, T., Lawson, D.J., Eichstaedt, C.A., Yngvadottir, B., Shwe, M.T., Wee, J., Romero, I.G., Raj, S., et al. (2014). Genome-wide analysis of cold adaptation in indigenous Siberian populations. PLoS One *9*, e98076.

Chen, T.W., Wardill, T.J., Sun, Y., Pulver, S.R., Renninger, S.L., Baohan, A., Schreiter, E.R., Kerr, R.A., Orger, M.B., Jayaraman, V., et al. (2013). Ultrasensitive fluorescent proteins for imaging neuronal activity. Nature *499*, 295–300.

Gehrig, S.M., van der Poel, C., Sayer, T.A., Schertzer, J.D., Henstridge, D.C., Church, J.E., Lamon, S., Russell, A.P., Davies, K.E., Febbraio, M.A., et al. (2012). Hsp72 preserves muscle function and slows progression of severe muscular dystrophy. Nature *484*, 394–398.

Grarup, N., Andersen, G., Krarup, N.T., Albrechtsen, A., Schmitz, O., Jorgensen, T., Borch-Johnsen, K., Hansen, T., and Pedersen, O. (2008). Association testing of novel type 2 diabetes risk alleles in the JAZF1, CDC123/CAMK1D, TSPAN8, THADA, ADAMTS9, and NOTCH2 loci with insulin release, insulin sensitivity, and obesity in a population-based sample of 4,516 glucose-tolerant middle-aged Danes. Diabetes *57*, 2534–2540.

Green, R.E., Krause, J., Briggs, A.W., Maricic, T., Stenzel, U., Kircher, M., Patterson, N., Li, H., Zhai, W., Fritz, M.H., et al. (2010). A draft sequence of the Neanderthal genome. Science *328*, 710–722.

Gupta, V., Vinay, D.G., Sovio, U., Rafiq, S., Kranthi Kumar, M.V., Janipalli, C.S., Evans, D., Mani, K.R., Sandeep, M.N., Taylor, A., et al. (2013). Association study of 25 type 2 diabetes related loci with measures of obesity in Indian sib pairs. PLoS One *8*, e53944.

Hanak, P., and Jezek, P. (2001). Mitochondrial uncoupling proteins and phylogenesis—UCP4 as the ancestral uncoupling protein. FEBS Lett. *495*, 137–141.

Huang, J., Zhou, W., Watson, A.M., Jan, Y.N., and Hong, Y. (2008). Efficient ends-out gene targeting in *Drosophila*. Genetics *180*, 703–707.

Hulbert, A.J., Clancy, D.J., Mair, W., Braeckman, B.P., Gems, D., and Partridge, L. (2004). Metabolic rate is not reduced by dietary-restriction or by lowered insulin/IGF-1 signalling and is not correlated with individual lifespan in *Drosophila melanogaster*. Exp. Gerontol. *39*, 1137–1143.

Kaufman, R.J., and Malhotra, J.D. (2014). Calcium trafficking integrates endoplasmic reticulum function with mitochondrial bioenergetics. Biochim. Biophys. Acta *1843*, 2233–2239.

Loli, D., and Bicudo, J.E. (2005). Control and regulatory mechanisms associated with thermogenesis in flying insects and birds. Biosci. Rep. *25*, 149–180.

Nan, C., Guo, B., Warner, C., Fowler, T., Barrett, T., Boomsma, D., Nelson, T., Whitfield, K., Beunen, G., Thomis, M., et al. (2012). Heritability of body mass index in pre-adolescence, young adulthood and late adulthood. Eur. J. Epidemiol. *27*, 247–253.

Naukkarinen, J., Rissanen, A., Kaprio, J., and Pietilainen, K.H. (2012). Causes and consequences of obesity: the contribution of recent twin studies. Int. J. Obes. *36*, 1017–1024.

Park, J., Kim, Y., Choi, S., Koh, H., Lee, S.H., Kim, J.M., and Chung, J. (2010). *Drosophila* Porin/VDAC affects mitochondrial morphology. PLoS One *5*, e13151.

Reis, M., Farage, M., de Souza, A.C., and de Meis, L. (2001). Correlation between uncoupled ATP hydrolysis and heat production by the sarcoplasmic reticulum Ca^{2+}-ATPase: coupling effect of fluoride. J. Biol. Chem. *276*, 42793–42800.

Sanghera, D.K., Been, L., Ortega, L., Wander, G.S., Mehra, N.K., Aston, C.E., Mulvihill, J.J., and Ralhan, S. (2009). Testing the association of novel meta-analysis-derived diabetes risk genes with type II diabetes and related metabolic traits in Asian Indian Sikhs. J. Hum. Genet. *54*, 162–168.

Sanyal, S., Consoulas, C., Kuromi, H., Basole, A., Mukai, L., Kidokoro, Y., Krishnan, K.S., and Ramaswami, M. (2005). Analysis of conditional paralytic mutants in *Drosophila* sarco-endoplasmic reticulum calcium ATPase reveals novel mechanisms for regulating membrane excitability. Genetics *169*, 737–750.

Schindelin, J., Rueden, C.T., Hiner, M.C., and Eliceiri, K.W. (2015). The ImageJ ecosystem: an open platform for biomedical image analysis. Mol. Reprod. Dev. *82*, 518–529.

Schleinitz, D., Tonjes, A., Bottcher, Y., Dietrich, K., Enigk, B., Koriath, M., Scholz, G.H., Bluher, M., Zeggini, E., McCarthy, M.I., et al. (2010). Lack of significant effects of the type 2 diabetes susceptibility loci JAZF1, CDC123/CAMK1D, NOTCH2, ADAMTS9, THADA, and TSPAN8/LGR5 on diabetes and quantitative metabolic traits. Horm. Metab. Res. *42*, 14–22.

Sellayah, D., Cagampang, F.R., and Cox, R.D. (2014). On the evolutionary origins of obesity: a new hypothesis. Endocrinology *155*, 1573–1588.

Simonis-Bik, A.M., Nijpels, G., van Haeften, T.W., Houwing-Duistermaat, J.J., Boomsma, D.I., Reiling, E., van Hove, E.C., Diamant, M., Kramer, M.H., Heine, R.J., et al. (2010). Gene variants in the novel type 2 diabetes loci CDC123/CAMK1D, THADA, ADAMTS9, BCL11A, and MTNR1B affect different aspects of pancreatic beta-cell function. Diabetes *59*, 293–301.

Staiger, H., Machicao, F., Kantartzis, K., Schafer, S.A., Kirchhoff, K., Guthoff, M., Silbernagel, G., Stefan, N., Fritsche, A., and Haring, H.U. (2008). Novel meta-analysis-derived type 2 diabetes risk loci do not determine prediabetic phenotypes. PLoS One *3*, e3019.

Stancakova, A., Kuulasmaa, T., Paananen, J., Jackson, A.U., Bonnycastle, L.L., Collins, F.S., Boehnke, M., Kuusisto, J., and Laakso, M. (2009). Association of 18 confirmed susceptibility loci for type 2 diabetes with indices of insulin release, proinsulin conversion, and insulin sensitivity in 5,327 nondiabetic Finnish men. Diabetes *58*, 2129–2136.

Subramanian, M., Jayakumar, S., Richhariya, S., and Hasan, G. (2013a). Loss of IP3 receptor function in neuropeptide secreting neurons leads to obesity in adult *Drosophila*. BMC Neurosci. *14*, 157.

Subramanian, M., Metya, S.K., Sadaf, S., Kumar, S., Schwudke, D., and Hasan, G. (2013b). Altered lipid homeostasis in *Drosophila* InsP3 receptor mutants leads to obesity and hyperphagia. Dis. Model. Mech. *6*, 734–744.

Teleman, A.A., Chen, Y.W., and Cohen, S.M. (2005). 4E-BP functions as a metabolic brake used under stress conditions but not during normal growth. Genes Dev. *19*, 1844–1848.

Wieckowski, M.R., Giorgi, C., Lebiedzinska, M., Duszynski, J., and Pinton, P. (2009). Isolation of mitochondria-associated membranes and mitochondria from animal tissues and cells. Nat. Protoc. *4*, 1582–1590.

World Health Organization. (2015). Global Health Observatory (GHO) Data (World Health Organization).

Ye, R., Ni, M., Wang, M., Luo, S., Zhu, G., Chow, R.H., and Lee, A.S. (2011). Inositol 1,4,5-trisphosphate receptor 1 mutation perturbs glucose homeostasis and enhances susceptibility to diet-induced diabetes. J. Endocrinol. *210*, 209–217.

Yogev, S., Schejter, E.D., and Shilo, B.Z. (2010). Polarized secretion of *Drosophila* EGFR ligand from photoreceptor neurons is controlled by ER localization of the ligand-processing machinery. PLoS Biol. *8*, e1000505.

Zeggini, E., Scott, L.J., Saxena, R., Voight, B.F., Marchini, J.L., Hu, T., de Bakker, P.I., Abecasis, G.R., Almgren, P., Andersen, G., et al. (2008). Meta-analysis of genome-wide association data and large-scale replication identifies additional susceptibility loci for type 2 diabetes. Nat. Genet. *40*, 638–645.

STAR★METHODS

KEY RESOURCES TABLE

REAGENT or RESOURCE	SOURCE	IDENTIFIER
Antibodies		
Anti-dTHADA guinea-pig polyclonal	This paper	N/A
Anti-dSERCA rabbit polyclonal	(Sanyal et al., 2005)	N/A
Anti-Calnexin rabbit polyclonal	ENZO	Cat#ADI-SPA-860-D; RRID: AB_2038898
Anti-Tubulin mouse monoclonal	Hybridoma	Cat#AA4.3-s; RRID: AB_579793
Anti-dPorin rabbit polyclonal	(Park et al., 2010)	RRID: AB_2569127
Anti-Glo1 rabbit polyclonal	Santa Cruz	Cat#sc-67351 lot no G0208; RRID: AB_1124969
anti-dLaminC	Hybridoma Bank	Cat#ADL 101-s; RRID: AB_528332
Chemicals, Peptides, and Recombinant Proteins		
Erioglaucine disodium salt	Sigma	Cat#861146
Complete protease inhibitor cocktail	Roche	Cat#15728900
PhosStop phosphatase inhibitor cocktail	Roche	Cat#04906837001
Lipoprotein lipase from Chromobacterium viscosum	Calbiochem	Cat#437707
Free Glycerol Reagent	Sigma	Cat#F6428
amyloglucosidase	Sigma	Cat#10115
glucose reagent	Sigma	Cat#G3293
porcine trehalase	Sigma	Cat#T8778
digitonin	Sigma	Cat#D141
phosphoenolpyruvate	Sigma	Cat#P7127
pyruvate kinase	Sigma	Cat#P1903-1KU
lactate dehydrogenase	Sigma	Cat#59747
calcimycin A-23187	Sigma	Cat#C7522
NADH	Sigma	Cat#N8129
ATP disodium salt	Sigma	Cat#A3377
Thapsigargin	BioVision	Cat#1558-1
Bodipy 493/503	Invitrogen	Cat#D3922
CellMask	Invitrogen	Cat#C10046
Oil Red O	Sigma	Cat#O1516
Schneider's Medium	GIBCO	Cat#21720
Ionomycin	SIGMA	Cat#I3909
Effectene	QIAGEN	Cat#301427
Lipofectamine RNAi Max	Invitrogen	Cat#13778150
Critical Commercial Assays		
Fluo4AM	Molecular Probes	Cat#F10489
Experimental Models: Cell Lines		
D. melanogaster: Cell line Kc167	Laboratory of Michael Boutros	N/A
D. melanogaster: Cell line S2R+	Laboratory of M. Schaefer	N/A
Human HeLa	Laboratory of Michael Boutros	N/A
Experimental Models: Organisms/Strains		
D. melanogaster THADA KO1	This paper	N/A
D. melanogaster THADA KO2	This paper	N/A
D. melanogaster UAS-dTHADA	This paper	N/A
D. melanogaster UAS-THADA-RNAi	This paper	N/A
D. melanogaster UAS-hTHADA	This paper	N/A

(*Continued on next page*)

Continued

REAGENT or RESOURCE	SOURCE	IDENTIFIER
D. melanogaster w[*]; P{w[+mC]=UASp-Ggal\LYZ.GFP.KDEL}401/CyO	Bloomington *Drosophila* Stock Center	BDSC: 31423; FlyBase: FBst0031423
D. melanogaster w[*]; P{w[+mC]=UAS-Grasp65-GFP}2	Bloomington *Drosophila* Stock Center	BDSC: 8507; FlyBase: FBst0008507
D. melanogaster y[1] w[*]; P{w[+mC]=UASp-YFP.Rab5}02	Bloomington *Drosophila* Stock Center	BDSC: 24616; FlyBase: FBst0024616
D. melanogaster UAS-FLAG-Serca	(Bi et al., 2014)	N/A
D. melanogaster P{UAS-Itp-r83A.V}1, w*	(Subramanian et al., 2013b)	FlyBase: FBst0030742
D. melanogaster UAS-SercaRNAi P{KK107371}VIE-260B	Vienna *Drosophila* Resource Center	v107446; FlyBase: FBst0479267
Oligonucleotides		
Cloning and gene expression knockdown oligonucleotides	This paper	Table S2
Recombinant DNA		
pUAS-FLAG-dSERCA	From Huang Xun lab (Bi et al., 2014)	N/A
pUAST-humanTHADA	This paper	N/A
THADA KO constructs #1 and #2	This paper	N/A
pMT-GCaMP6s	This paper	N/A
CMV-GCaMP6s	Addgene	Cat#40753
Software and Algorithms		
ImageJ2	(Schindelin et al., 2015)	N/A
Digitam	Thermometric AB	N/A
Other		
Affinity based mass spectrometry data	This paper	Table S1

CONTACT FOR REAGENT AND RESOURCE SHARING

Further information and requests for resources and reagents should be directed to and will be fulfilled by the Lead Contact, Aurelio Teleman (a.teleman@dkfz.de).

EXPERIMENTAL MODEL AND SUBJECT DETAILS

Experimental Animals

Species

Drosophila melanogaster. Flies were grown and maintained on food consisting of the following ingredients for 30 liters of food: 480g agar, 660g sugar syrup, 2400g malt, 2400g corn meal, 300g soymeal, 540g yeast, 72g nipagin, 187mL propionic acid and 18.7 mL phosphoric acid.

Flies carrying GFP-KDEL (31423), Grasp65-GFP (8507), and YFP-Rab5 (24616) were from the Bloomington *Drosophila* Stock Center. SercaRNAi flies were from the Vienna *Drosophila* Resource Center (v107446). FLAG-Serca flies were a kind gift from Dr. Xun Huang, described in (Bi et al., 2014). UAS-IP3R (M31) flies were a kind gift from Prof. Gaiti Hasan, described in (Subramanian et al., 2013a).

Both male and females were used, as indicated in the figure legends.

Human Cell Lines

HeLa cells were maintained in high-glucose DMEM medium (Thermo Fisher Scientific) supplemented with 10% FBS (Biochrom). Identity of the HeLa cells was verified by the Multiplex human Cell line Authentication test (MCA, Multiplexion), which uses an SNP-profiling approach as described at www.multiplexion.de. The cells were verified to be free of Mycoplasma, or contamination with cells of other species, according to the Multiplex cell Contamination Test Report (Multiplexion).

Drosophila Cell Lines

Drosophila melanogaster cell lines Kc167 and S2R+ were maintained in Schneider's medium (GIBCO 21720) supplemented with Penicillin/Streptomycin and 10%FBS.

METHOD DETAILS

Generation of THADA Knockout and Rescue Flies

THADA^{KO1} and THADA^{KO2} flies were generated by cloning 4kb upstream and downstream genomic flanking sequences, using the oligos described in Table S2, into the AvrII-AscI and NheI-NotI sites of the pRK1 vector (Huang et al., 2008), respectively. Knockout

flies were generated as described in (Huang et al., 2008). For KO1, this yielded a 1024nt deletion covering the 4th exon and the beginning of the 5th exon, and causing the remainder of the open reading frame to be out-of-frame. For KO2, this yielded a 1910nt deletion which includes the knockout region of KO1, but extends to include the transcription start site and basal promoter of THADA. The genetic background of THADA^{KO} flies was cleaned by 4 generations of backcrossing to an isogenic w^{1118} stock, which is used as the control stock in all experiments described here.

The rescue construct used to generate UAS-dTHADA animals was generated by cloning the genomic sequence of THADA into pBluescript, sequencing to identify a clone with no mutations, and subsequent subcloning into pUAST.

Fly Rearing Conditions for Metabolic Analyses

For all growth and metabolic measurements, flies were grown under controlled conditions. Flies were allowed to lay eggs on apple plates for 12 hours. After an additional 11 hour incubation, plates were cleared of hatched first instar larvae. First instar larvae hatching within the subsequent 4 or 6 hour window were picked and seeded at a density of 50 or 60 animals per vial, depending on the experiment. Adult flies of all genotypes eclosing within a 24-hour time-window were then chosen for the experiment, and aged an equal number of days (between 3 and 7 days, depending on the experiment).

Food Intake, TAG and Glycogen Measurements

For rescue experiments, food intake, TAG content and glycogen content of flies were measured simultaneously using a combined assay: Flies were kept on fly food supplemented with 1%(w/v) Acid Blue 9 (erioglaucine disodium salt, Sigma 861146), females flies for 3 hours and male flies for 1 hour. Triplicates of 8 flies per sample were then washed once with PBST (0.05% (v/v) Tween 20 in PBS), and homogenized in 500 μl of PBST using a table-top drill with a plastic pestell. 300 μl of lysate was incubated at 70°C for 5 min, chilled on ice, and incubated with 1μL of 25KU/mL Lipoprotein lipase from Chromobacterium viscosum (Calbiochem, cat. 437707) at 37°C overnight. Cellular debris were removed by centrifugation at 14000rpm for 3 min. Food intake was quantified by measuring absorbance of 200 μl of supernatant at 625nm. TAG content was determined by mixing 15 μl of the supernatant with 150 μl Free Glycerol Reagent (Sigma F6428), incubated at 37°C for 6 min and absorbance was measured at 540nm. The background absorption due to the food color was determined for each sample by measuring absorbance of 15 μl of the supernatant mixed with 150 μl water, and then subtracting it from the TAG measurement. Data were normalized to total protein measured by Bradford assay at 595nm. For determining the glycogen content 30 μl of the supernatant (after LPL treatment) was treated with 14Units of amyloglucosidase (Sigma 10115) at 50°C for 1hour. 15 μl of the treated mix was combined with 150 μl of glucose reagent (Sigma G3293), incubated at 37°C for 30 min and absorbance was measured at 340nm. These data were also normalized to total protein.

When only the TAG and glycerol content were measured, flies were kept on standard food, and homogenized in PBST and processed as described in the combined assay above.

Pupation Curves

Fifty first-instar larvae were seeded per vial, six vials for each genotype, and grown under controlled conditions, in standard food at 25C as described above. Third instar larvae were separated by gender and reseeded into three vials for each. The number of pupated animals was counted over time, and is represented as a percentage of total pupated animals.

Starvation Resistance

4 days old adult female flies, grown under controlled conditions as described above, were transferred from normal food to 1% agarose/PBS and dead animals were counted over time.

Wing Size and Cell Size

Adult animals were grown under controlled conditions as described above, and then fixed in 70% ethanol/ 30% glycerol overnight. Wings were mounted in Hoyer’s medium on glass slides, imaged, and analyzed using ImageJ. The entire wing area was used to determine wing size. To determine cell size, the number of trichomes (which are made one per cell) were counted in two areas of fixed size per wing – one in the anterior and one in the posterior compartments – and averaged together. Cell size was calculated by dividing the area by the number of cells in that area.

Lifespan

Animals were raised in controlled growth conditions (50 larvae per vial), and co-aged adults were then kept at 25 degrees in horizontally-positioned vials. Animals were transferred into fresh vials every 3 days to prevent molding.

Circulating Trehalose & Glucose Measurements

Hemolymph was collected from 8 wandering third instar larvae into TBS pH6.6 (137mM NaCl, 2.7mM KCl, 5mM Tris pH6.6), followed by heat-inactivation at 70 °C for 5 min. Half of the lysate was then incubated with porcine trehalase (Sigma T8778) at 37 °C overnight and half was kept without the enzyme to measure the levels of free glucose. Total glucose and free glucose were then measured using a colorimetric kit from Sigma (G3293).

Serca Activity Assay

Ca2+-dependent SERCA activity was measured in whole-lysates by a spectrophotometric assay using an enzyme-coupled system as previously described (Gehrig et al., 2012). Flies were homogenized in Homogenization buffer (250mM sucrose, 5mM HEPES pH7.0, 1mM PMSF, complete protease inhibitor cocktail (Roche)). The homogenization buffer for lysing cells contained in addition 25 μM digitonin. Protein concentration was adjusted with Homogenization buffer to 140 μg/mL (for fly lysates) or 25 μg/mL (for cell lysates), and added into reaction buffer (100mM KCl, 10 mM MgCl2, 20 mM HEPES, pH 7.0, 10 mM phosphoenolpyruvate, 1 mM EGTA, 15 U/ml each of pyruvate kinase and lactate dehydrogenase, 0.5 mM NADH, 2 μM calcimycin A-23187 (C7522 Sigma), 5μM free Ca^2, final pH 7.0) Reactions were started by adding 5mM ATP, incubated for 10 min at 37 °C, then the decrease in absorbance at 340nm was recorded for 10 min. SERCA-independent Ca2+-ATPase activity was measured in the presence of the SERCA inhibitor Thapsigargin (10 μM) (BioVision 1558-1) and subtracted.

Microcalorimetry

The rate of heat production was measured following the protocol in (Hulbert et al., 2004) using 2277 Thermal Activity Monitors and Digitam software (Thermometric AB). Growth controlled flies were placed in groups of five in a recipient containing 1cm2 Whattman paper soaked with 80μL distilled water. The rate of heat produced at 25°C was determined in the dark during three hours following 30 min calorimeter equilibration. Heat production rate was calculated as the average over the three hours of measurement. After the measurement the flies were weighed and the amount of heat was normalized to body weight.

Cold Sensitivity Recovery Assay

Growth controlled one week old flies were exposed to 4°C for 3 hours. Afterwards the vials were kept at RT to allow recovery of the flies. The recovery of the flies was inspected by eye and the number of flies climbing was recorded every two minutes.

Bodipy Staining

Adult female abdomens were opened in PBS, and gut and reproductive organs were removed. The carcass with attached fat body were immersed and imaged in 1x PBS containing 10 μM Bodipy 493/503 (Invitrogen D3922) to visualise lipid droplets and 5 μg/ml CellMask (Invitrogen C10046) to mark cell membranes.

Oil Red Staining

Adult female guts were fixed in 4% paraformaldehyde/PBS for 30 min at room temperature. Guts were then rinsed twice with PBS, incubated for 30 min in Oil red staining solution (6 ml of 0.1% Oil Red O in isopropanol and 4 ml distilled water, prepared fresh and rinsed through a 0.45 μM syringe filter) and then rinsed twice with distilled water. Guts were imaged in mounting medium (160 ml glycerol, 20 ml 10x PBS, 12 ml water, 0,8 g n-propyl gallate).

Antibody Production

To generate anti-drosophila THADA antibody, a His-tagged N-terminal fragment of the THADA protein was produced by amplifying the coding sequence corresponding to amino acids 75-457 into the pETM11 plasmid, and expressing it in a bacterial host. Purified, recombinant protein was then used to immunize guinea pigs.

Other Antibodies

Rabbit anti-SERCA antibody was a kind gift from Dr. Sanyal Subhabrata, described in (Sanyal et al., 2005). Anti-Calnexin (ENZO ADI-SPA-860-D) anti–Tubulin (Hybridoma AA4.3-s), anti-dPorin (a kind gift from Dr. Jongkyeong Chung, described in (Park et al., 2010), anti-dLaminC (Hybridoma Bank ADL 101-s), anti-Glo1 1:1000 (Santa Cruz sc-67351 lot no #G0208).

Immunostainings

Larval tissues were dissected in cold 1xPBS and fixed in 4% paraformaldehyde in 1xPBS for 20 min at RT. The samples were washed three times with 1xPBS/TritonX-100 0.2%, then blocked for 45 minutes in 1xPBS/ TritonX-100 0.2%/BSA 0.1%. Primary antibody was incubated overnight at 4°C (anti dTHADA 1:200, anti dSERCA 1:500 in blocking buffer), followed by four washes of 30 min each with 1xPBS/TritonX-100 0.2%. The secondary fluorescent antibodies were incubated for two hours at RT in blocking solution. After washing the samples four times with 1xPBS, the tissues were mounted in mounting medium (80% glycerol, 0.4% N-propyl-gallate, 1x PBS). The slides were imaged with a Leica SP8 confocal microscope.

Cell Culture and dsRNA Treatments

Drosophila cells were cultured at 25°C in Schneider's Medium (GIBCO 21720) supplemented with Penicillin/Streptomycin and 10% FBS. dsRNA was generated by in vitro transcription with T7 RNA polymerase using as templates PCR products which were generated using the oligos listed in Table S2. Gene knockdowns were performed by treating cells with 12ug/ml dsRNA in serum-free medium for one hour, followed by 5 days of growth in complete medium to allow for protein depletion.

Plasmid Transfection

Plasmid DNA transfections in *Drosophila* cells were done with Effectene (QIAGEN), according to manufacturer's instructions.

GCaMP6 Fluorescent Assays

For GCaMP6 fluorescent assays in *Drosophila* cells, Kc167 or S2R+ cells were transfected overnight with an inducible pMT-GCaMP6 plasmid (the GCaMP6 ORF was cloned into pAT1250 via BglII+NotI restriction sites) followed by overnight induction with copper. Cells were attached to poly-L-lysine coated wells for imaging (Ibidi 80821) and the medium was replaced with Schneider's supplemented with 10 mM EGTA prior to addition of 2μM Ionomycin (SIGMA I3909) or 2μM Thapsigargin (BioVision 1558-1) Time-lapse images were recorded with a Leica SP8 confocal microscope. The GCaMP6 fluorescent signal is represented as the average of relative change in fluorescence intensity normalized to baseline intensity (ΔF/F0). The quantification was performed using ImageJ2.

For GCaMP6 assays in mammalian cells, HeLa cells were seeded in 4-well IbiTreat coated imaging wells (Ibidi 80426) at the density of 50000 cells/well. The next day they were transfected with siRNA (pool of four siRNAs from Dharmacon D-032022) using Lipofectamine RNAi Max (Invitrogen), according to the manufacturer's protocol. The second day they were transfected with a CMV-GCaMP6s expression plasmid (Addgene Plasmid #40753) (Chen et al., 2013) using Effectene (QIAGEN), according to the manufacturer's protocol. After 48 hours the cells were imaged. The medium was replaced with DMEM supplemented with 2mM EGTA just prior to addition of 2μM Thapsigargin (BioVision 1558-1). Time-lapse images were recorded with a Leica SP8 confocal microscope. The GCaMP6 fluorescent signal was represented as the average of relative change in fluorescence intensity normalized to baseline intensity (ΔF/F0). The quantification was performed using ImageJ2.

Fluo4AM Staining

Drosophila Kc167 and S2 cells that have been treated for 5 days with dsRNA were attached to poly-L-lysine coated wells (Ibidi 80821). Fluo4AM mix was prepared according to the manufacturer's protocol (Molecular Probes F10489), and incubated with the cells for one hour at RT. Afterwards the wells were washed once and the medium was replaced with fresh Schneider's medium. The chambers were imaged with a Leica SP8 confocal microscope using the FITC settings and the quantification of the Fluo4AM signal per cell was performed using ImageJ2.

Immunoprecipitation

Flies or cells were lysed in IP lysis buffer (50mM Tris pH7.5, 1%Triton-X, 150mM NaCl, 1.5x Complete protease inhibitor cocktail without EDTA, 2x PhosStop phosphate inhibitor cocktail, 4mM Na vanadate, 100mM Na fluoride, 11mg/mL beta-glycerophosphate), cleared by centrifugation, incubated with indicated antibody for 3 hours at 4°C, followed by incubation with protein A beads for 30 minutes at 4°C, then washed with IP buffer 4 times. The beads were boiled in reducing Laemmli buffer and analyzed by Western blotting or mass spectrometry.

Microsome Isolations

Microsomes were isolated by differential centrifugation as previously described (Bi et al., 2014; Gehrig et al., 2012). Thirty wandering third instar larvae were homogenized in 1.5mL homogenization buffer (250mM sucrose, 5mM HEPES pH7.0, 1mM PMSF, complete protease inhibitor cocktail (Roche)) on ice, followed by centrifugation at 1,000g for 10 min at 4 °C. The supernatant was centrifuged 2 times at 12,000g for 15 min at 4°C, followed by centrifugation for 60 min at 100,000g in a Beckman Optima Max ultracentrifuge at 4 °C. The final pellet, containing enriched microsomes was resuspended in homogenization buffer and boiled in reducing Laemmli buffer.

Crude Mitochondrial Preparations

Isolation of crude mitochondria was adapted from (Wieckowski et al., 2009). 24 flies were homogenized in 300 uL of IB-1 buffer (225-mM mannitol, 75-mM sucrose, 0.5% BSA, 0.5-mM EGTA and 30-mM Tris–HCl pH 7.4) and centrifuged for 5 minutes 750g at 4 C. The pellet containing nuclei and unbroken cells was homogenized in 375 uL of 1xLaemmli buffer. The supernatant was centrifuged again for 5 minutes 750g at 4 C, then for 10 minutes 9000g at 4 C. The resulting supernatant containing cytosol, plasma membrane, lysosomes and microsomes, was boiled after addition of 74 uL of 5xLaemmli. The remaining pellet was resuspended in 1.5 mL of ice-cold IB-2 buffer (225-mM mannitol, 75-mM sucrose, 0.5% BSA and 30-mM Tris–HCl pH 7.4) and centrifuged for 10 minutes 10000g at 4 C. The resulting pellet containing crude mitochondria was resuspended in 200 uL of 1x Laemmli.

Human THADA cDNA Cloning

The Human THADA open reading frame was cloned from cDNA obtained from HEK293 cells in three fragments. The first fragment was cloned using primers OAM155+OAM156 and digested with BglII and NcoI, the second fragment was cloned using primers OAM157+OAM158 and digested with NcoI and AgeI and the third fragment was cloned using primers OAM159+OAM160 and digested with AgeI and NotI. The three fragments were cloned into a pUAST vector linearized with BglII and NotI in a four piece ligation reaction.

QUANTIFICATION AND STATISTICAL ANALYSIS

Quantifications

Total body triglycerides and total body glycogen were normalized to total body protein, as described in the section " Food intake, TAG and Glycogen measurements". Wing size was quantified by measuring the area of imaged wings using ImageJ, and cell size

was quantified by counting the number of trichomes in an area of fixed size, and dividing the area by the number of trichomes, as detailed in the section ''Wing Size and Cell Size''. Heat production was normalized to fly weight as described in the section ''Microcalorimetry''.

Statistical Analyses

The statistical comparisons for mutants versus controls were done by Student t-test. All details of statistical analyses, including n values, are found in the figure legends.

Developmental Cell, Volume *41*

Supplemental Information

THADA Regulates the Organismal Balance between Energy Storage and Heat Production

Alexandra Moraru, Gulcin Cakan-Akdogan, Katrin Strassburger, Matilda Males, Sandra Mueller, Markus Jabs, Michael Muelleder, Martin Frejno, Bart P. Braeckman, Markus Ralser, and Aurelio A. Teleman

Supplemental Information

Inventory

1. Supplemental Figures

- Figure S1. (Related to main Figure 1).
- Figure S2. (Related to main Figure 1).
- Figure S3. (Related to main Figure 1).
- Figure S4. (Related to main Figure 2).
- Figure S5. (Related to main Figure 4).

2. Legends to Supplemental Figures

3. Supplemental Tables

- Table S1. (Related to main Figure 2).
- Table S2. (Related to STAR Methods).

Moraru et al. - Supplemental Figure 1

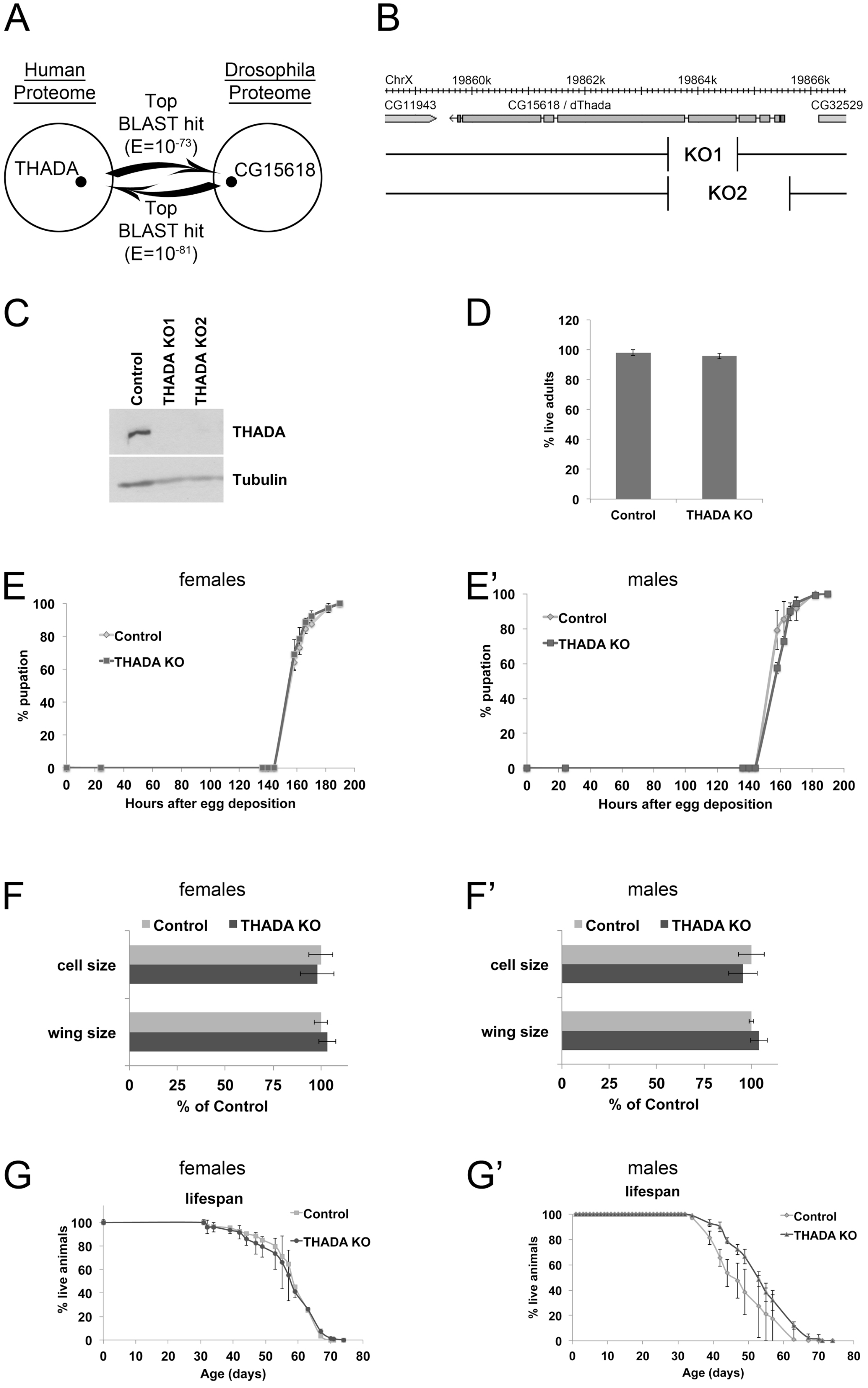

Moraru et al. - Supplemental Figure 2

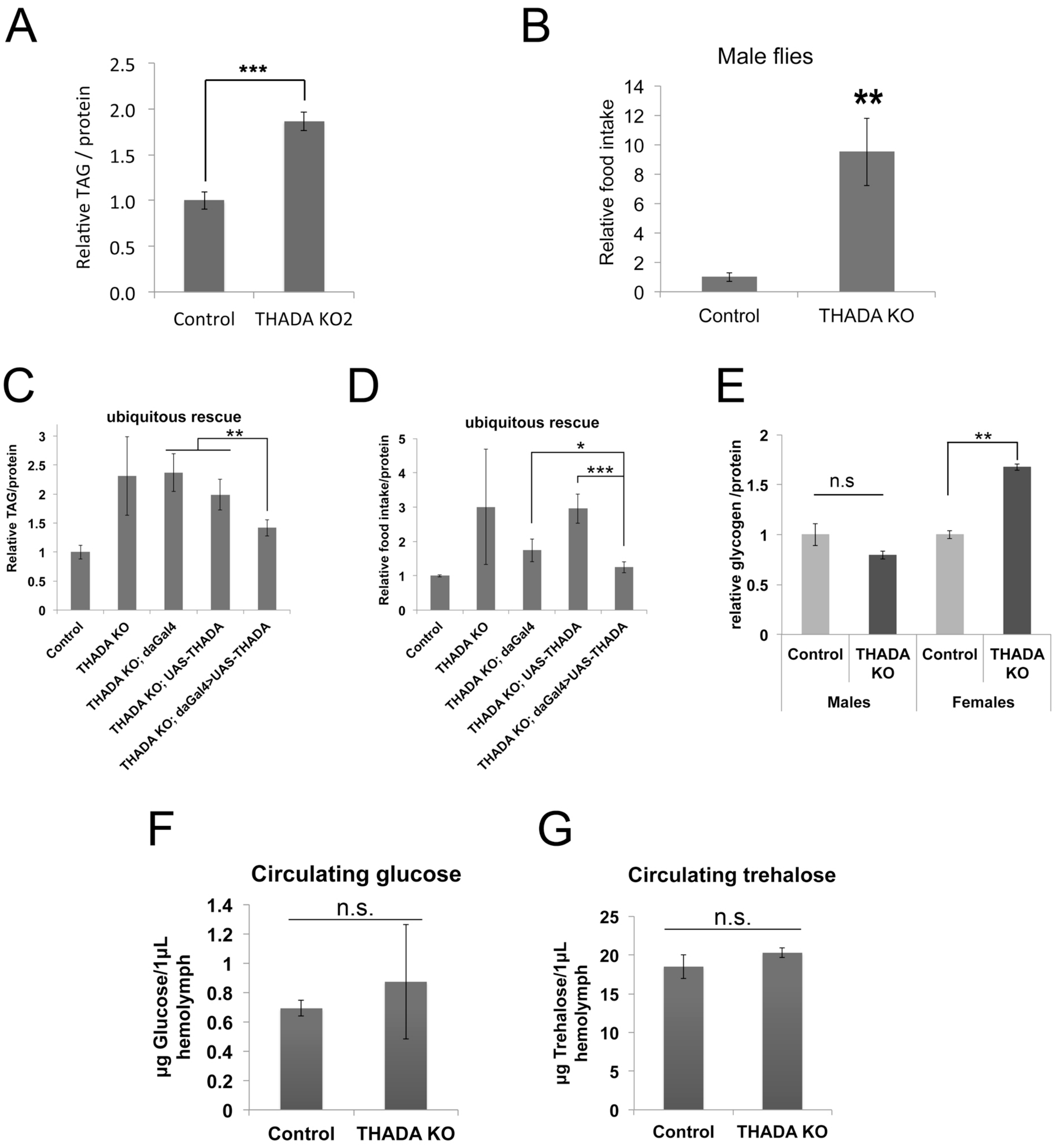

Moraru et al. - Supplemental Figure 3

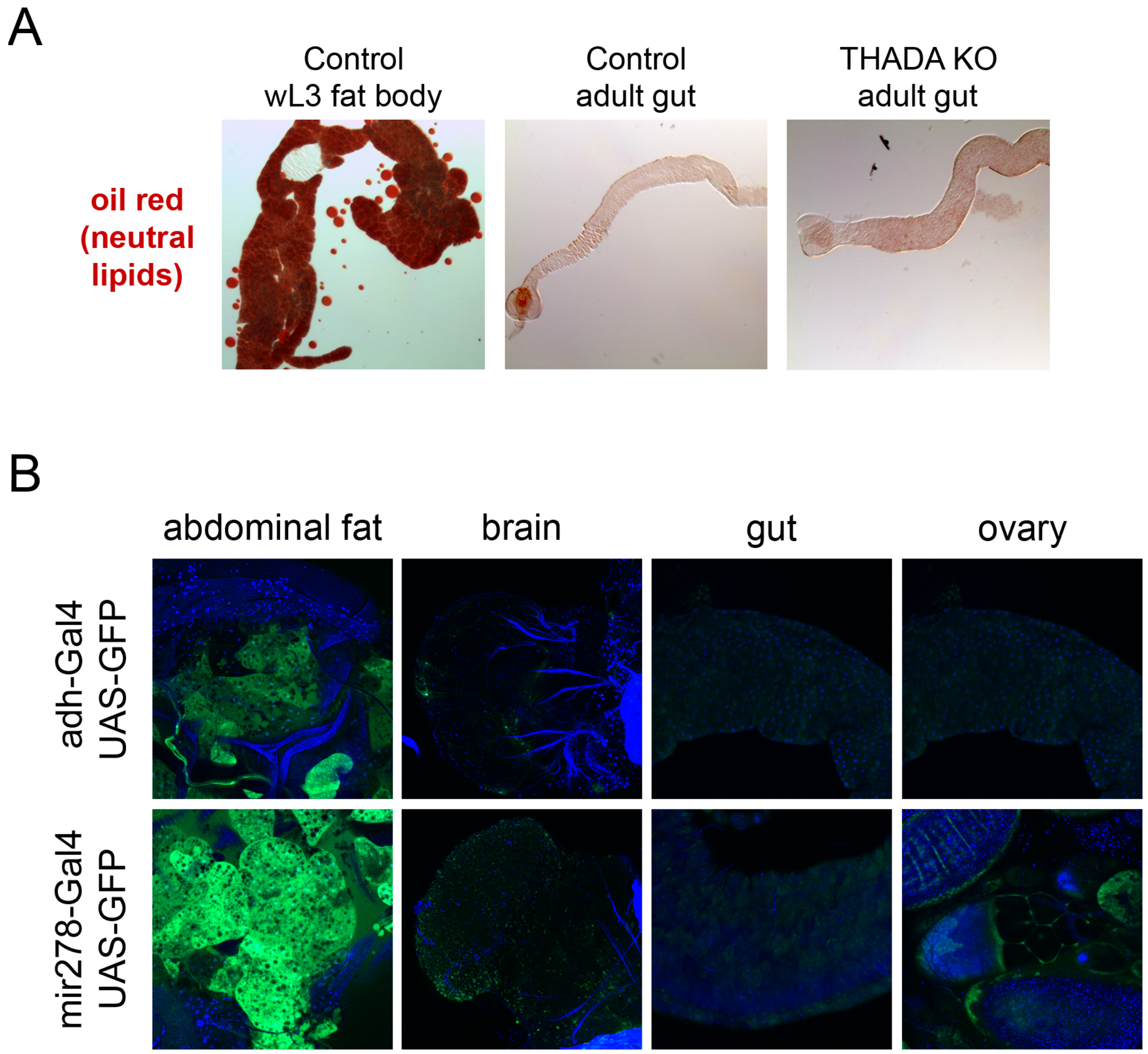

Moraru et al. - Supplemental Figure 4

A

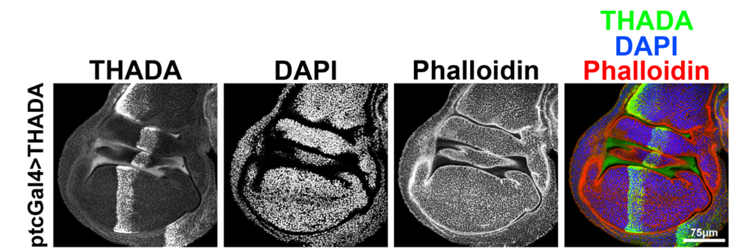

B

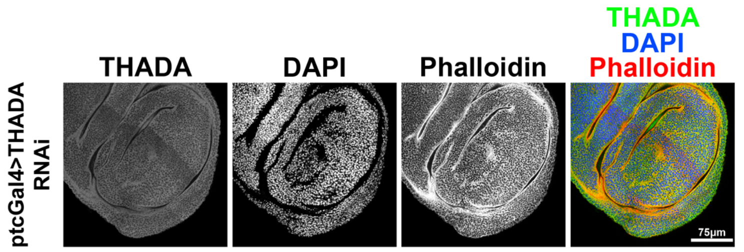

C

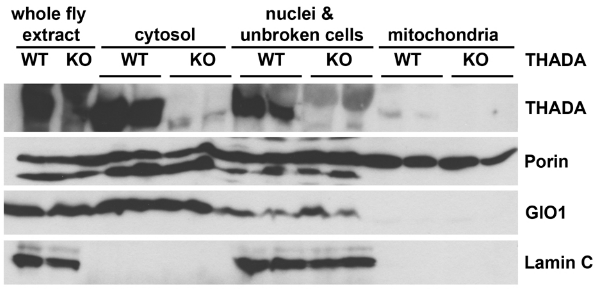

Moraru et al. - Supplemental Figure 5

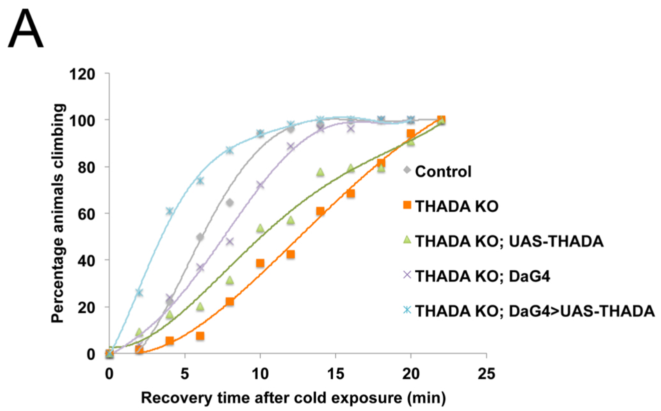

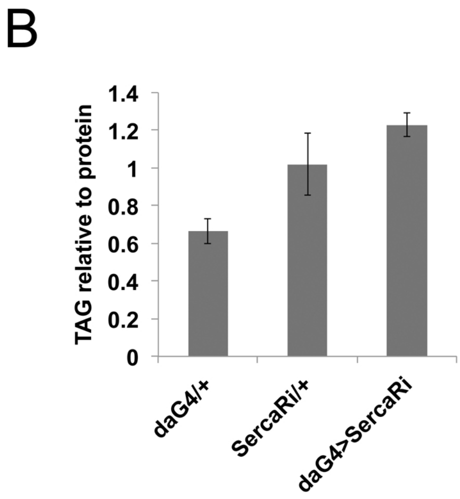

Legends to Supplemental Figures

Figure S1: *Drosophila THADA* knockout flies are viable. Related to Figure 1.

(A) CG15618 is the Drosophila ortholog of THADA. BLAST search (using the Flybase BLAST server) of the Drosophila proteome using the protein sequence of human THADA (NCBI Reference Sequence NP_071348.3) identifies CG15618 as the top hit, with an E value of 10^{-73}. Conversely, a BLAST search of the human proteome using the protein sequence of Drosophila CG15618 identifies THADA as the top hit with an E value of 10^{-81}, establishing an orthologous relationship between human THADA and Drosophila CG15618, which we henceforth refer to as Drosophila THADA.

(B) Schematic diagram of the *THADA/CG15618* genomic locus, indicating the knock-out regions.

(C) Anti-dTHADA antibody detects a band at the expected 220kDa size in control flies, but not in $THADA^{KO1}$ or $THADA^{KO2}$ flies.

(D-F') *THADA* knockout flies are viable (D), and have normal developmental timing (E-E') and size (F-F'). Sixty first-instar larvae were seeded per vial, and grown under controlled conditions. (D) Total number of eclosing adults was counted, and normalized to number of seeded larvae (n=3x60 animals). (E-E') Number of pupated females (E) or males (E') was quantified over time, represented as a percentage of total pupated animals (n=3x40 animals). (F-F') Total wing area and wing cell size (area per number of wing hairs) were measured and normalized to control animal values (n=8, representative of 3 experiments) for females (F) and males (F').

(G-G') *THADA*^{KO} flies have normal longevity. Lifespan curves of *THADA*^{KO} females (G) and males (G') are similar to those of controls. (n=4 x 35 to 55 adult females)

Figure S2: *Drosophila THADA* knockout flies have metabolic defects. Related to Figure 1.

(A) *THADA*^{KO2} animals, like *THADA*^{KO1} animals, have strongly elevated total body triglyceride levels, normalized to total body protein content. (n=3x8 3-day old adult females).

(B) *THADA*^{KO} males (like females, shown in Main Figure 1) are hyperphagic. Relative food intake in *THADA*^{KO} adult male flies, quantified by transferring flies from normal food to food containing Acid Blue 9 for one hour, followed by homogenization and measurement of OD625. (n=3x9)

(C-D) Ubiquitous expression of *THADA* from a transgene rescues the elevated triglycerides (C) and the hyperphagia (D) of *THADA*^{KO} flies. (C) Total body triglycerides normalize to total body protein. (D) Food intake quantified as in Main Figure 1B. Expression of *THADA* was achieved using the GAL4/UAS bipartite system whereby the GAL4 transcription factor activates expression downstream of a *UAS* enhancer element. Parental genotypes containing the *THADA* mutation and either the *GAL4* or *UAS-THADA* do not express *THADA*, whereas the combination (*THADA*^{KO}; *daughterless(da)-GAL4>UAS-THADA*) restores *THADA* expression. Note that the hyperphagia phenotype (D) is more variable than the obesity phenotype (C) since the

hyperphagia phenotype in some genetic backgrounds (e.g. *THADA*^{KO}; *da-GAL4*) is not strong. (n=3x9 adults females per genotype)

(E) *THADA*^{KO} females, but not males, have elevated glycogen levels. Total body glycogen levels in adult flies, normalized to total body protein. (n=3x8 adult flies per genotype)

(F-G) *THADA*^{KO} animals do not have significantly altered circulating sugar levels. Circulating glucose (F) and trehalose (G) were determined from hemolymph isolated from wandering third instar female larvae. (n=3x8)

Error bars: Std. dev. *p<0.05, **p<0.01, ***p<0.001, "n.s." p=0.47(F) or 0.13(G) by student t-test.

Figure S3: THADA knockout flies do not have ectopic lipid deposition. Related to Figure 1.

(A) Oil Red staining for neutral lipids reveals little to no ectopic lipid deposition in THADA^{KO} guts, a typical organ for ectopic lipid deposition in Drosophila.

(B) Adh-GAL4 and mir278-GAL4 are predominantly expressed in the adult fat body. Expression of the two GAL4 drivers revealed by crossing to UAS-GFP and imaging GFP fluorescence. Images of all tissues for one genotype were taken with the same laser and confocal settings.

Figure S4: Specificity controls for anti-THADA antibody and lack of strong THADA signal in mitochondrial preparations. Related to Figure 2.

(A-B) Control immunostainings of wing discs with α-dTHADA antibody show that it specifically detects THADA protein. Wing imaginal discs expressing either *UAS-THADA* to overexpress *THADA* (A) or *THADA RNAi* to reduce expression of endogenous *THADA* (B) in a central stripe of the wing disc using the *patched-Gal4* driver.

(C) Only very low levels of THADA protein are found in preparations of crude mitochondria from flies. Porin serves as a control for mitochondria, Lamin C for nuclei, and Glyoxalase I for cytosol.

Figure S5: Support to main Figure 4.

(A) Sensitivity to cold exposure of *THADA*^{KO} flies is rescued by ubiquitous expression of *THADA* from a UAS transgene. Adult flies were kept for 5 hours at 4°C to immobilize them. Recovery at room temperature was monitored as the ability to resume climbing. Parental genotypes containing the *THADA* mutation and either the ubiquitous *daughterless-GAL4* (*DaG4*) or *UAS-THADA* do not express THADA, whereas the combination (*THADA*^{KO}*; DaGAL4>UAS-THADA*) restores *THADA* expression. Sensitivity curve of the rescued animals is significantly different compared to *THADA*^{KO}*;DaG4 animals* (Log-rank test p-score <0.0001). (n=3x18 per genotype)

(B) Ubiquitous expression of SERCA-RNAi in control flies does not reduce their total body triglyceride levels. Total body triglyceride levels normalized to

total body protein for adult female flies with a mild, ubiquitous SERCA knockdown using *daughterless-GAL4* (*daG4>SercaRi*), compared to the parental control genotypes carrying either the GAL4 only or the *UAS-SERCA-RNAi* only. (Error bars: Std. Dev. n=3 x 8).

Supplemental Tables

Table S1: THADA interacting proteins. (Related to Figure 2).

Available as an excel sheet for download from the website.

Table S2: Cloning and gene expression knockdown Oligonucleotides. (Related to STAR Methods)

Oligo Name	Purpose	Sequence
GCA001	Clone upstream flank for THADA knockout #1 construct (AvrII)	ccggcctaggGGGCGGTTGTCGAGGATGTG
GCA004	Clone upstream flank for THADA knockout #1 construct (AscI)	ccggggcgcgccGCATTGTGCGCAGCTATCAT
GCA005	Clone downstream flank for THADA knockout #1 construct (NheI)	ccgggctagcGCCGCCAGGTCATTGAGGAG
GCA008	Clone downstream flank for THADA knockout #1 construct (NotI)	ccgggcggccgcGCTGTGCGCATGGAGAGATC
OAM099	clone downstream flank	TATGGGCGCACTGGGTTAGCAGA

	for THADA knockout #2	
OAM100	clone downstream flank for THADA knockout #2	actagtTTCAGATGGGCGAAGAAATCGAT
GCA360	dsRNA1 against THADA CG15618	ggcctaatacgactcactatagggaggATCCGCAATCCTGACAGAAC
GCA 361	dsRNA1 against THADA CG15618	ggcctaatacgactcactatagggaggGTTGGCGGACACGAAGAC
GCA362	dsRNA2 against THADA CG15618	ggcctaatacgactcactatagggaggGGGCCAGGAACTGTATGA
GCA363	dsRNA2 against THADA CG15618	ggcctaatacgactcactatagggaggAGCCTGATGGCGAAGATA
OAM155	part 1 hThada upper	agatctCCGACGTGCACGAGTGACTACT
OAM156	part 1 hThada lower	ACTCCTGGAGACTGGCTGTTAA
OAM157	part 2 hThada upper	GGGAGAACCTCGTGTCTGATGC
OAM158	part 2 hThada lower	GCACTGCCCAGACCGGTGATGT
OAM159	part 3 hThada upper	AGATACGCGCCTGGGAGAAAAT
OAM160	part 3 hThada lower	gcggccgcCCCCATCCCAATCCCCCAGATT
THADA siRNA pool	Dharmacon	Cat#D-032022
